# Supplementary material for: Wind power density in areas of Northeastern Brazil from Regional Climate Models for a recent past
Source: PLoS One. 2024 Jul 25;19(7):e0307641. doi: 10.1371/journal.pone.0307641 (PMC11271907; doi:10.1371/journal.pone.0307641)

PLOS ONE

Wind power density in areas of Northeastern Brazil from regional climate models for a recent past

--Manuscript Draft--

**Manuscript Number:**	PONE-D-24-02304
**Article Type:**	Research Article
**Full Title:**	Wind power density in areas of Northeastern Brazil from regional climate models for a recent past
**Short Title:**	Wind power density in areas of Northaestern Brazil
**Corresponding Author:**	Augusto de Rubim Costa Gurgel<br>Federal University of Rio Grande: Universidade Federal do Rio Grande<br>Natal, BRAZIL
**Keywords:**	Regional Climate Models; ECMWF-ERA5 reanalysis; Wind power density; Northeastern Brazil.
**Abstract:**	Investments in renewable energy sources are being made in several countries, especially in wind energy, due to global climate change caused by the burning of fossil fuels to generate electricity. Thus, it's important to know the Regional Climate Models that promote the best model of wind speed and wind power density in promising areas for generating this type of energy in the recent past, essential for the implementation of wind farms. Therefore, the general objective of this research is to calculate the wind power density from Regional Climate Models in areas in the Northeast of Brazil from 1986 to 2005. To this end, initially, the reanalysis of the ECMWF-ERA5 was validated in relation to the data observed from Xavier. The results were satisfactory, with moderate correlations of 0.4 to 0.69 and bias between -1m/s and 1m/s. Then, the Regional Climate Models RegCM4.7, RCA4 and Remo2009 were validated with the ECMWF-ERA5 reanalysis, which managed to represent well the wind speed pattern, but overestimated this variable in the Northeast of Brazil, especially during the dry season of the region. In addition, four areas were chosen in the Northeast of Brazil for study. In these areas, the simulations of the models that presented the best statistical indices for each chosen area were validated and selected: RegCM4.7_HadGEM2 (northern coastal of Ceará and northern coastal of Rio Grande do Norte) and RCA4_Miroc (Borborema and Central Bahia). After the selection of the models, the wind power density was calculated in each area of the Northeast of Brazil, as a result, the area of North of Rio Grande do Norte presented the highest density of wind power.
**Order of Authors:**	Augusto de Rubim Costa Gurgel
	Domingo Cassain Sales
	Kellen Carla Lima
**Additional Information:**	
**Question**	**Response**
**Financial Disclosure**<br><br>Enter a financial disclosure statement that describes the sources of funding for the work included in this submission. Review the submission guidelines for detailed requirements. View published research articles from *PLOS ONE* for specific examples.<br><br>This statement is required for submission	The author(s) received no specific funding for this work.

Powered by Editorial Manager® and ProduXion Manager® from Aries Systems Corporation

and **will appear in the published article** if the submission is accepted. Please make sure it is accurate.<br><br>**Funded studies**<br>Enter a statement with the following details:<br>• Initials of the authors who received each award<br>• Grant numbers awarded to each author<br>• The full name of each funder<br>• URL of each funder website<br>• Did the sponsors or funders play any role in the study design, data collection and analysis, decision to publish, or preparation of the manuscript?<br><br>Did you receive funding for this work?	
**Competing Interests**<br><br>Use the instructions below to enter a competing interest statement for this submission. On behalf of all authors, disclose any competing interests that could be perceived to bias this work—acknowledging all financial support and any other relevant financial or non-financial competing interests.<br><br>This statement is required for submission and **will appear in the published article** if the submission is accepted. Please make sure it is accurate and that any funding sources listed in your Funding Information later in the submission form are also declared in your Financial Disclosure statement.<br><br>View published research articles from *PLOS ONE* for specific examples.	The authors have declared that no competing interests exist.

**NO authors have competing interests**<br><br>Enter: *The authors have declared that no competing interests exist.*<br><br>**Authors with competing interests**<br><br>Enter competing interest details beginning with this statement:<br><br>*I have read the journal's policy and the authors of this manuscript have the following competing interests: [insert competing interests here]*<br><br>* typeset	
**Ethics Statement**<br><br>Enter an ethics statement for this submission. This statement is required if the study involved:<br><br>• Human participants<br>• Human specimens or tissue<br>• Vertebrate animals or cephalopods<br>• Vertebrate embryos or tissues<br>• Field research<br><br>Write "N/A" if the submission does not require an ethics statement.<br><br>General guidance is provided below. Consult the submission guidelines for detailed instructions. **Make sure that all information entered here is included in the Methods section of the manuscript.**	N/A

**Format for specific study types**<br><br>**Human Subject Research (involving human participants and/or tissue)**<br>• Give the name of the institutional review board or ethics committee that approved the study<br>• Include the approval number and/or a statement indicating approval of this research<br>• Indicate the form of consent obtained (written/oral) or the reason that consent was not obtained (e.g. the data were analyzed anonymously)<br><br>**Animal Research (involving vertebrate animals, embryos or tissues)**<br>• Provide the name of the Institutional Animal Care and Use Committee (IACUC) or other relevant ethics board that reviewed the study protocol, and indicate whether they approved this research or granted a formal waiver of ethical approval<br>• Include an approval number if one was obtained<br>• If the study involved *non-human primates*, add *additional details* about animal welfare and steps taken to ameliorate suffering<br>• If anesthesia, euthanasia, or any kind of animal sacrifice is part of the study, include briefly which substances and/or methods were applied<br><br>**Field Research**<br><br>Include the following details if this study involves the collection of plant, animal, or other materials from a natural setting:<br>• Field permit number<br>• Name of the institution or relevant body that granted permission	
**Data Availability**<br><br>Authors are required to make all data underlying the findings described fully available, without restriction, and from the time of publication. PLOS allows rare exceptions to address legal and ethical concerns. See the PLOS Data Policy and FAQ for detailed information.	Yes - all data are fully available without restriction

Powered by Editorial Manager® and ProduXion Manager® from Aries Systems Corporation

A Data Availability Statement describing where the data can be found is required at submission. Your answers to this question constitute the Data Availability Statement and **will be published in the article**, if accepted.<br><br>**Important:** Stating 'data available on request from the author' is not sufficient. If your data are only available upon request, select 'No' for the first question and explain your exceptional situation in the text box.<br><br>Do the authors confirm that all data underlying the findings described in their manuscript are fully available without restriction?	
**Describe where the data may be found in full sentences. If you are copying our sample text, replace any instances of XXX with the appropriate details.**<br><br>• If the data are **held or will be held in a public repository**, include URLs, accession numbers or DOIs. If this information will only be available after acceptance, indicate this by ticking the box below. For example: *All XXX files are available from the XXX database (accession number(s) XXX, XXX.).*<br>• If the data are all contained **within the manuscript and/or Supporting Information files**, enter the following: *All relevant data are within the manuscript and its Supporting Information files.*<br>• If neither of these applies but you are able to provide **details of access elsewhere**, with or without limitations, please do so. For example:<br><br>*Data cannot be shared publicly because of [XXX]. Data are available from the XXX Institutional Data Access / Ethics Committee (contact via XXX) for researchers who meet the criteria for access to confidential data.*<br><br>*The data underlying the results presented in the study are available from (include the name of the third party*	The datasets analyzed during the current study are available in the:<br><br>Xavier observed data is available in: https://utexas.app.box.com/v/Xavier-etal-IJOC-DATA<br><br>ECMWF-ERA5 reanalysis data is available in: https://cds.climate.copernicus.eu/cdsapp#!/dataset/reanalysis-era5-single-levels-monthly-means?tab=form<br><br>Regional Climate Models data from CORDEX-CORE are available in: https://esgf-index1.ceda.ac.uk/search/cordex-ceda/

and contact information or URL).<br>• This text is appropriate if the data are owned by a third party and authors do not have permission to share the data.<br><br>* typeset	
Additional data availability information:	

Powered by Editorial Manager® and ProduXion Manager® from Aries Systems Corporation

Manuscript
Click here to access/download;Manuscript;Artigo_plos_one_Augusto_Kellen

Wind power density in areas of Northeastern Brazil from regional climate models for a recent past

Augusto de Rubim Costa Gurgel[1*], Domingo Cassain Sales[2&], Kellen Carla Lima[1&]

[1] Postgraduate Program in Climate Science, Center for Exact and Earth Sciences, Federal University of Rio Grande do Norte, Natal, Rio Grande do Norte, Brazil
[2] Fundação Cearense de Meteorologia e Recursos Hídricos, Fortaleza, CE, Brazil.

*Corresponding Author
E-mail: rubim10@yahoo.com.br (ARCG)
ORCID ID: http://orcid.org/0000-0002-0581-9475

[&]These authors also contributed equally to this work

Abstract

Investments in renewable energy sources are being made in several countries, especially in wind energy, due to global climate change caused by the burning of fossil fuels to generate electricity. Thus, it's important to know the Regional Climate Models that promote the best model of wind speed and wind power density in promising areas for generating this type of energy in the recent past, essential for the implementation of wind farms. Therefore, the general objective of this research is to calculate the wind power density from Regional Climate Models in areas in the Northeast of Brazil from 1986 to 2005. To this end, initially, the reanalysis of the ECMWF-ERA5 was validated in relation to the data observed from Xavier. The results were satisfactory, with moderate correlations of 0.4 to 0.69 and bias between -1m/s and 1m/s. Then, the Regional Climate Models RegCM4.7, RCA4 and Remo2009 were validated with the ECMWF-ERA5 reanalysis, which managed to represent well the wind speed pattern, but overestimated this variable in the Northeast of Brazil, especially during the dry season of the region. In addition, four areas were chosen in the Northeast of Brazil for study. In these areas, the simulations of the models that presented the best statistical indices for each chosen area were validated and selected: RegCM4.7_HadGEM2 (northern coastal of Ceará and northern coastal of Rio Grande do Norte) and RCA4_Miroc (Borborema and Central Bahia). After the selection of the models, the wind power density was calculated in each area of the Northeast

of Brazil, as a result, the area of North of Rio Grande do Norte presented the highest density of wind power.

Key Words: Regional Climate Models. ECMWF-ERA5 reanalysis. Wind power density. Northeast Brazil.

Introduction

Knowledge about wind speed and its local variability is fundamental, as they are essential elements to carry out an economic evaluation of the development of wind farm projects [1]. Environmental issues are being raised due to the impacts of climate change from the burning of fossil fuels to produce electricity nowadays. In this way, technologies aimed at wind energy are being developed rapidly because it is considered a clean source of energy, helping countries to find new alternatives to produce electricity, with lower Greenhouse Gases emissions (GHG) [2,3].

In Brazil, the production of electricity generated by renewable sources exceeds the production by non-renewable sources. According to the International Renewable Energy Agency [4], in 2021, 83.9% of the energy generated in the country came from renewable sources, ~~while 16.1% came from non-renewable sources~~. However, one of the main problems faced by Brazil is its dependence on hydroelectric power, which in 2021 accounted for 53.55% of the country's energy production. Due to the lack of rainfall, the Northeast region of Brazil (NEB) faced a period of very extensive drought, which began in 2012 [5], affecting the production of hydroelectric energy, requiring the use of thermoelectric energy. Thus, diversification in energy production is necessary, and, in Brazil, wind energy emerges as a potential source that has been following global growth. In the last five years, its production in Brazil jumped from 33,240.1 GWh in 2016 to 71,499.9 GWh in 2021. In 2021, generation accounted for 11.22% of the country's total energy production [4].

The NEB is the Brazilian region with the greatest potential for wind development. Due to its geographical position, the region benefits from the trade winds that are intense, stable and blow in

the same direction most of the time [6]. All the states in this region have a border with the Atlantic Ocean, so that the wind speed on this border is higher because it does not present barriers. In particular, the states of Ceará, Rio Grande do Norte and Bahia are the ones that produce the largest amounts of wind energy in the country [7].

Wind speed can be measured by anemometers at any height, however measurements higher than 10m in height are hardly found, since high investments are necessary for structural reasons [8]. One of the ways to overcome this challenge is the use of mathematical methods, such as logarithmic and exponential functions [9,10]. In addition to these issues, weather stations have flaws in their measurements and large spacing between them, resulting in low-quality data to enable wind projects [11].

In the face of these difficulties, climatic reanalysis products are used by researchers to study wind speed. The reanalysis presents historical series assimilated by control models based on meteorological variables available by some type of observation [12]. Recently, the European Centre for Medium-Range Weather Forecast (ECMWF) presented the ECMWF-ERA5 reanalysis, combining large amounts of historical observations into estimates for the globe. This dataset has coverage for the period from 1950 to the present with regular grid resolution of 0.25° × 0.25° (latitude x longitude) and 137 levels of vertical surface pressure [13]. Many studies use data from reanalysis to validate data observed in climate change studies [11,14,15].

In the field of climate change, the Coordinated Regional Climate Downscaling Experiment (CORDEX-CORE) [16] provides data from research centers that use Regional Climate Models (RCM) in future climate projections. These rounds include the period called the recent past (historical experiment) and simulations for the future (up to 2100). RCMs are fed by boundary conditions of the Global Climate Models (GCM) belonging to the Coupled Model Intercomparison Project – Phase 5 (CMIP5) [17]. From the dynamic downscaling technique, the RCMs are able to capture regional climatic characteristics, such as: extreme weather events, seasonal variations in precipitation,

orographic precipitation and simulate characteristics of climatic anomalies that impact on a regional scale such as El Niño - Southern Oscillation (ENSO) [18].

The validation of the reanalysis with the observed data is important to evaluate the accuracy of these data, however, due to the lack of interpolated data, the validations occur in a punctual manner. In addition, validating the models in the recent past is important for the climate simulations of the future. However, model validation studies for wind speed and wind power density using climate models belonging to CORDEX-CORE and the Representative Concentration Pathways (RCP) are few for the NEB and for South America as a whole.

In South America, [10] used ECMWF-ERA-Interim reanalysis data to validate the RCM RegCM4 and evaluate the projection of wind speed for the near (2020-2050) and distant (2070-2098) future, in the RCP8.5 scenario, at 10 and 100 m from ground height. The authors observed a reduction in wind speed in regions in the Northeast and Midwest of Brazil during the austral summer, as well as an increase in this variable in all regions of Brazil in the other seasons of the year, both for the near and distant future.

To the authors' knowledge, there are no previous studies that have validated the spatial ECMWF-ERA5 reanalysis for the NEB. In addition, previous studies focused on South America have validated only a limited amount of RCM, generally focusing only on RegCM4 and not considering RCA4 and Remo2009 RCAs. In addition, the high climatic diversity presented by the NEB associated with the lack of emphasis in the study of wind speed behavior in areas with a large number of installed wind farms and the high resolution of the RCM motivated this study.

Thus, the general objective of the research is to calculate the wind power density from Regional Climate Models in areas of Northeast Brazil for the recent past from 1986 to 2005. Specifically, it is intended to: (i) spatially validate the ECMWF-ERA5 reanalysis for the NEB in comparison with the observed Xavier data; (ii) validate the RCMs for the NEB in relation to the reanalysis; (iii) Identify the RCMs with the best statistical indices for the NEB areas.

Section 2 describes the study area, the observed data, reanalysis and RCM used for the validations, as well as the methodology applied to calculate the wind power density. Section 3 presents the results of the reanalysis and RCM validations, wind variability in NEB areas, as well as the choice of the model with the best metrics for calculating wind power density. Finally, section 4 reports the findings of this research.

Materials ~~and methods~~

Study area

The NEB is between the meridian 48°05' W and 35°02' W and the parallels 1°S and 18°05'S. The region is composed of nine Brazilian states: Maranhão (MA), Piauí (PI), Ceará (CE), Rio Grande do Norte (RN), Paraíba (PB), Pernambuco (PE), Alagoas (AL), Sergipe (SE) and Bahia (BA) (Fig 1). Four study areas were chosen in the NEB region, considering the number of wind farms installed and the different climatic aspects. Herein, N-CE (red): northern coastal region of Ceará; N-RN (black): northern coastal region of Rio Grande do Norte; Borborema (yellow): the region of the Borborema plateau that covers the states of Rio Grande do Norte, Paraíba and the wild landscape (agreste) of Pernambuco, and finally, C-BA (green): comprises the region of central Bahia.

Fig 1. Location of the NEB in Brazil with wind farms in activity and study areas.

Data

Xavier Observed Data

The monthly observational database chosen as a reference to validate the wind speed of the ECMWF-ERA5 reanalysis was made available by [19], which here was denominated observed data.

This database has several climatic variables, but here, the authors used only the intensity of wind speed at 2m for the period from 1986 to 2005, interpolated at horizontal grid points of high resolution 0.25°×0.25° at latitude-longitude for all of Brazil. This data was extrapolated to 10m for comparison with the data from the ECMWF-ERA5 reanalysis. The observed data interpolated in the same grid as the reanalysis allowed to validate the reanalysis spatially in the NEB, ensuring the use of the reanalysis, which covers wide spatial and temporal coverage, in addition to presenting wind speed data for various altitudes, throughout the research.

ECMWF-ERA5 Reanalysis data

The monthly reanalysis ECMWF-ERA5 data was available in Copernicus Climate Data Store. Wind speed data at 10 m from the ground were used to validate the observed data. After validation, wind speed at 100 m from the ground was used to validate the RCM data. In addition, the variability of wind speed at 100 m above the ground was evaluated in the NEB areas adopted in this study. All data present monthly frequency, within the period between 1986 and 2005 and a grid with regular horizontal spatial resolution of 0.25° in latitude and longitude. The horizontal spatial grid was maintained for comparison purposes with the observed data, however, they were interpolated to a horizontal spatial grid of 0.44° in latitude and longitude for comparison with the RCM.

CORDEX-CORE Regional Climate Models

This study was performed from nine models from CORDEX-CORE. The wind speed data at 100 m ground height of the RCMs were used for validation with ECMWF-ERA5 reanalysis. The data show monthly frequency, the period used was the recent past (1986-2005) and the horizontal spatial grid of 0.44° was used in latitude and longitude. Then, the RCM data of wind speed at 100 m height from the ground were compared in areas of the NEB for the period of the recent past (1986-2005),

aiming to validate the models with the best statistical indices in each study area. Then, the best wind speed data at 100 m ground height model were used for wind speed analysis and wind power density calculation in the recent past (1986-2005).

The RCM RegCM4.7 features a regular horizontal spatial resolution of 0.22° in latitude and longitude, while the RCA4 and Remo2009 models feature a grid with a regular horizontal spatial resolution of 0.44° in latitude and longitude. The wind speed of the RCMs, RCA4 and Remo2009 are only available at 10m from the ground. Thus, for the purpose of comparing the models, RegCM4.7 was interpolated to the horizontal spatial resolution grid of 0.44° in latitude and longitude and the RCA4 and Remo2009 data were extrapolated to 100 m height. Table 1 specifies the RCMs as well as the global models used as large-scale forcings.

Table 1 RCMs belonging to CORDEX-CORE and their global forcings

RCM·(Country) ¤	Resolution¤	GCM·(Country) ¤	Resolution¤
RegCM4.7·(Italy) · [20] ¤	0,22°·×·0,22° ¤	HadGEM2-ES·(UK)·[23] ¤	1,2°·×·1,9°¤
		NorESM1-M·(Norway)·[24] ¤	2,5°·×·1,9°¤
		MPI-ESM-MR·(Germany)·[25] ¤	1,9°·×·1,9°¤
RCA4· (Sweden)·[21] ¤	0,44°·×·0,44° ¤	HadGEM2-ES·(UK)·[23] ¤	1,2°·×·1,9°¤
		NorESM1-M·(Norway)·[24] ¤	2,5°·×·1,9°¤
		MPI-ESM-LR·(Germany)·[25] ¤	1,9°·×·1,9°¤
		EC-EARTH·(International)·[26] ¤	1,1°·×·1,1°¤
		Miroc5·(Japan)·[27] ¤	1,4°·×·1,4°¤
Remo2009· (Germany)·[22] ¤	0,44°·×·0,44° ¤	MPI-ESM-LR·(Germany)·[25] ¤	1,9°·×·1,9°¤

Methodology

Extrapolation of wind speed to 10m and 100m in the comparison between databases.

The comparison between the data needs to be carried out at the same height. Thus, the wind speed of the observed data was extrapolated from 2 to 10 m in order to validate the ECMWF-ERA5 reanalysis. In addition, the data from the RCA4 and Remo2009 models were extrapolated from 10 to 100m for comparison with the ECMWF-ERA5 reanalysis. The extrapolation occurred by logarithmic law expressed [28]:

$$U(z) = U(zr)\frac{ln\left(\frac{Z}{Z_0}\right)}{ln\left(\frac{Z_r}{Z_0}\right)} \quad (1)$$

Where: U(z) = wind speed at the desired height, U(Z_r) = wind speed relative to the ground, Z_0 = surface roughness, Z = desired height, Z_r = height of the die relative to the ground. For each grid point of the model, the surface roughness was estimated based on the table provided in the RegCM4 manual [29].

Validations: Reanalysis versus Observation and Regional Climate Models versus NEB Reanalysis.

Initially, in order to measure the accuracy of the ECMWF-ERA5 reanalysis in relation to the data observed in the NEB for the period of the recent past (1986-2005), analyses were performed based on the following statistical metrics: bias and Pearson's correlation coefficient. This validation occurred for seasonal averages with the wind speed variable at 10 m from the ground. Then, the seasonal variability of wind speed at 100 m from the ground throughout the NEB is shown for reanalysis and the RCMs data is validated by bias, identifying if the RCMs presents seasonal trends in relation to the reanalysis. The bias is expressed by:

$$bias = \frac{1}{n}\sum_{i=1}^{N} P_i \qquad (2)$$

Wherel, $P_i = P_{i,mod} - P_{i,obs}$ is the difference between the wind speed value of the ECMWF-ERA5 reanalysis ($P_{i,mod}$) and the value of this same variable from the observed data $P_{i,obs}$ at the same place and time (i = 1,2,3…,n), while n is the number of observations (for each quarter, there are 60 values because it is covering a period of 20 years). After the bias calculation, Student's t-tests were performed to determine the statistical significance of the results. A 95% confidence interval and two-tailed distribution were assumed [30].

$$t = \frac{\bar{P}_{obs} - \bar{P}_{mod}}{\sqrt{\left(\left(\frac{s_{obs}^2}{n_{obs}}\right) + \frac{s_{mod}^2}{n_{mod}}\right)}} \qquad (3)$$

Wherende: $\bar{P}_{obs}, \bar{P}_{mod}$ it's the averages; S_{obs} and S_{mod} are the standard deviations; and n_{obs} and n_{mod} are the number of elements in the time series. The "obs" index represents the observed data and the "mod" represents the reanalysis.

Pearson's correlation coefficient describes the degree of collinearity between simulated and observed data. This index ranges from -1 to 1, with the best results close to value 1 [31]. Table 2 presents a classification developed by [31]. The coefficient is mathematically expressed by:

$$r = \frac{\sum_{i=1}^{n}(P_{obs,i} - \bar{P}_{obs})(P_{mod,i} - \bar{P}_{mod})}{\sqrt{(\sum_{i=1}^{n} P_{obs,i} - \bar{P}_{obs})^2 x (\sum_{i=1}^{n} P_{mod,i} - \bar{P}_{mod})^2}} \qquad (4)$$

Where: $P_{obs,i}$ is the value observed data at time i; $\bar{P}_{obs}$ is the average observed data value; $P_{mod,i}$ is the estimated value of the reanalysis at time i; $\bar{P}_{mod}$ is the estimated mean value of the reanalysis; n is the number of observations; r is the correlation coefficient.

Table 2 Classification of Pearson's correlation coefficients.

Classification¤	r intervals
Very·weak¤	0,00·to·0,19¤
Weak¤	0,20·to·0,39¤

Moderate	0,40 to 0,69
Strong	0,70 to 0,89
Very strong	0,90 to 1,0

Source: [31].

Monthly variability of wind speed at 100 m from the ground in areas of the NEB.

In order to know the wind behavior in each study area of the NEB for the recent past from 1986 to 2005, the analysis of descriptive numerical measurements for the intensity of wind speed at 100m from the ground was carried out from the reanalysis of the ECMWF-ERA5. In this analysis, boxplots were constructed to verify details about the seasonal variation of wind speed: minimum recorded, 1st quartile, median, mean, 3rd quartile and maximum wind speed. In addition, variance and standard deviation were included.

Validation: Regional Climate Models versus Reanalysis in NEB areas.

RCMs were validated individually for the entire NEB compared to ECMWF-ERA5 reanalysis data. However, the NEB region is extensive and presents several active meteorological phenomena that diversify the climate of the region, in addition, the RCMs have high horizontal spatial resolution, suggesting that some models may present better results than others for some specific areas. Therefore, it is important to validate which models present the best statistical indexes for wind speed simulations in relation to the reanalysis in the areas with the largest number of installed wind farms, so that the wind power density of the respective area can be calculated with the least possible error.

The annual wind speed cycle between reanalysis and RCMs was evaluated for the NEB study areas in order to observe if the models could capture the wind speed pattern of the reanalysis. According to [18], one way to observe an improvement or additional knowledge among RCMs is

through the Taylor diagram. The Taylor's diagram [32] presents robust statistical metrics in a single diagram, including the Root of Mean Square Error (RMSE), Pearson's Correlation Coefficient, and standard deviation. The result of these indicators provides the performance of the models. The basic data for comparison is the wind speed of the reanalysis, so in the diagram the closer the model is to the reanalysis, the better the simulation of the model for the area. Since RMSE, defined mathematically to be used in Talor Diagram:

$$RMSE = \sqrt{S\,(mod) + S\,(obs) - 2 * S(mod) * S(obs) * r(mod, obs)} \quad (5)$$

Where: S(mod) and S(obs) is the standard derivation and r(mod,obs) is the Pearson's correlation coefficient between RCM and observed. The RMSE ranges from 0 to infinity, with 0 being the ideal result. Here "mod" is attributed to RCMs and "obs" is attributed to ECMWF-ERA5 reanalysis.

The standard deviation of the model should be the closest to the value of the observation. The standard deviation is calculated:

$$S = \sqrt{\frac{\left(P_{data,i} - \bar{P}_{data}\right)^2}{(n-1)}} \quad (6)$$

Where: $P_{data,i}$ is the stimulated value of the model/observed at time i; $\bar{P}_{data}$ is the average stimulated value of the model/observed; n is the number of observations; S is the standard deviation.

Wind power density from Regional Climate Models in NEB areas.

The wind power density of a given region estimates the amount of Watts of electrical energy produced per unit square meter (W/m^2). In this research, the calculation of the wind power density was performed from the monthly average of the specific mass of air and wind speed. The wind speed variable is extremely important in the calculation of the wind power density, due to a small inaccuracy of the value can generate a very large error because this is a square variable. Some studies use medium

ensembles [10,33] of models to reduce this inaccuracy, in this research, this variable was used based on the result of the best model for each study area and the objective is to show the best possible result. Mathematically, the equation is [34]:

$$P(v) = \frac{1}{2} \times \rho v^3 \qquad (7)$$

Where:ρ = specif mass of air; v = wind speed (m/s).

The specific mass of air, can be defined as [28]:

$$\rho = \frac{\left(353{,}4\left(1 - \frac{Z}{45271}\right)^{5{,}2624}\right)}{273{,}15} + T \quad (8)$$

Where: Z= height (m), T = Room temperature (°C)

The temperature was obtained for the CORDEX-CORE models at 2 m from the ground. To use these values at 100 m from the ground, a dry adiabatic laps rate was considered, in which every 100 m of height the temperature decreases by 1°C [35].

Results

Validation: Reanalysis versus Observation across the NEB.

Fig 2 shows the seasonal variability of Pearson's correlation coefficient (left) and bias (right) of wind speed intensity from the reanalysis versus data observed throughout the NEB for the recent past from 1986 to 2005.

Fig 2. Seasonal variability of Pearson's correlation and bias of wind speed, reanalysis versus observed data. (a) Correlation DJF, (b) bias DJF, (c) correlation MAM, (d) bias MAM, (e) correlation JJA, (f) bias JJA, (g) correlation SON, (h) bias SON

Regarding the austral summer (DJF), Pearson's correlation coefficient (Fig 2A), the northern region of the NEB shows a strong correlation with a predominance of values between 0.7 and 0.9. Part of the western sector of the NEB has the lowest values of Pearson's correlation coefficient, considered very weak and weak with values up to 0.4, while the southern region of the NEB has a Pearson’s correlation coefficient between very weak and strong, with values above 0.0 to 0.7. For the bias results in this season of the year (Fig 2B), the reanalysis shows absolute values lower than 1 m/s in most of the NEB.

In the following quarter, austral autumn (MAM – Fig 2C), reductions in Pearson's correlation coefficients are observed. In the northern region of the NEB, Pearson's correlation values are between 0.4 and 0.9, i.e., between moderate and strong. The central and southern regions of the NEB have a predominance of Pearson's correlation coefficient between 0.2 and 0.7, i.e., ranging from weak to moderate. As for the bias (Fig 2D), most of the NEB presents values in the range between -1 and 1m/s.

The season of the year with the highest values of Pearson's correlation coefficient is the austral winter (JJA – Fig 2E), when in almost all areas of the NEB they present values between moderate and strong (0.4 to 0.9), reaching the classification of very strong (between 0.9 and 1) in coastal areas of the northern part of the NEB. On the other hand, when analyzing the wind speed bias (Fig 2F), it is observed to be the station with the largest spatial area of overestimation of wind speed, with values up to 2 m/s for the central area of the NEB.

Finally, for the austral spring (SON – Fig. 2G), Pearson's correlation coefficient values are the lowest for the NEB, with values between 0.0 and 0.4, considered between very weak and weak on the northern coast of the NEB. In some areas south of the and center of the NEB, Pearson's correlation coefficients between moderate and strong, 0.4 to 0.9, are found. Fig 2H (bias) shows values for most of the NEB, ranging from -1 to 1 m/s. All of the results are statistical significant for t-test with 95% of significance.

Seasonal variability of wind speed at 100m from the ground across NEB.

Fig 3 shows the seasonal mean wind speed at 100 m from the ground for the ECMWF-ERA5 reanalysis in the recent past period from 1986 to 2005. Climatologically, the maximum velocities are observed in the JJA and SON quarters (Figs 3C and 3D, respectively), with mean velocities between 6 and 9 m/s in the northern coastal region (which comprises the states of MA to RN, as well as in the region of the Borborema plateau between the states of RN and PB, and the wild landscape (agreste) of PE, reaching the northern and central regions of BA.

For summer (DJF – Fig 3A) and austral autumn (MAM – Fig 3B), the coastal regions between MA and RN, the Borborema plateau between RN and PB and the PE wild landscape, in addition to the north of BA, present velocities ranging between 4 and 7 m/s. Despite lower speeds in these seasons, compared to the JJA and SON quarters, wind speeds still have a minimum necessary for wind energy production.

Fig 3. Seasonal average wind speed (m/s) at 100m from the reanalysis for the recent past (1986-2005): (a) DJF, (b) MAM, (c) JJA e (d) SON.

Validation: Regional Climate Models versus NEB-wide Reanalysis

The models used here will be given nomenclature based on the RCM and GCM. The name on the left refers to the RCM and on the right to the GCM that forces it globally, so they are called: RegCM4.7_HadGEM2, RCA4_HadGEM2, RegCM4.7_MPI, RCA4_MPI, Remo2009_MPI, RegCM4.7_NorESM, RCA4_NorESM, RCA4_Miroc, and RCA4_EARTH.

Fig 4 shows the bias of the wind speed intensity of the RCM versus reanalysis across the NEB for the austral summer (DJF) in the recent past period from 1986 to 2005. The RegCM4.7_HadGEM2

model (Fig 4A) is the only model that overestimates the intensity of wind speed for the NEB by 1 to 3 m/s.

Models RegCM4.7_MPI, RCA4_HadGEM2, RCA4_MPI, RCA4_EARTH, RCA4_miroc, and Remo2009_MPI (Figs 4B, 4D, 4E, 4G, 4H, and 4I) capture a similar pattern for wind speed. They overestimate the wind speed for the states of MA and PI with values between 1 and 3 m/s, with the models simulated by MCG MPI being more intense and with greater areas of overestimation. In addition, these models tend to underestimate the wind for the entire coastline of the NEB, with values between 1 and 3 m/s, except for the Remo2009_MPI. Models RegCM4.7_MPI, RCA4_MPI and RCA4_EARTH are the most intense and the ones with the greatest extent in underestimation.

The RegCM4.7_NorESM and RCA4_NorESM models (Figs 4C and 4F) capture similar patterns, where they underestimate the wind speed on the northern coastal coast of the NEB with values between 1.5 and 3 m/s and overestimate the east coast of the NEB, expanding to more central regions with values between 2 and 3 m/s. The RegCM4.7_NorESM tends to have more areas of overestimation of wind speed while the RCA4_NorESM has more areas of underestimation of wind speed.

Fig 4. Bias of RCM wind speed intensity versus NEB-wide reanalysis for the austral summer (DJF).

Fig 5 shows the wind speed intensity bias of the RCM versus reanalysis across the NEB for the austral autumn (MAM) in the recent past period from 1986 to 2005. The bias pattern of wind speed simulated by the RCM is similar to the pattern obtained in the DJF period.

The RegCM4.7_HadGEM2 model (Fig 5A) is the only model that continues to overestimate wind speed across the NEB but reduces positive bias errors compared to the previous quarter. It continues to overestimate the east coast of NEB and south-central BA with values between 1 and 2.5 m/s.

The RegCM4.7_MPI, RCA4_MPI, and Remo2009_MPI models (Figs 5B, 5E, and 5I) reduce the intensities and overestimated areas in MA and PI, especially the REMO2009_MPI. The area of underestimation of these models is reduced, RegCM4.7_MPI and RCA4_MPI underestimate the wind speed in the eastern part of the NEB to the center of the BA with values up to 2 m/s. For the RCA4_HadGEM2 and RCA4_EARTH models (Figs 5D and 5G), the bias for MA and PI is reduced to values below 1 m/s. Model RCA4_miroc (Fig 5H) shows intensification of overestimation of wind speed in the central and southern regions of the NEB with values between 1 and 3 m/s.

Models RegCM4.7_NorESM and RCA4_NorESM (Figs 5C and 5F) show intensification of wind speeds. The region of wind underestimation is reduced to the states of MA, PI and CE with values between 1 and 2.5 m/s. Model RegCM4.7_NorESM overestimates the wind speed throughout the state from PB to BA with values up to 3 m/s, for model RCA4_NorESM this overestimation is concentrated further west in PB extending to the south of BA with values between 1.5 and 3 m/s.

Fig 5. Bias of RCM wind speed intensity versus NEB-wide reanalysis for the austral autumn (MAM).

Fig 6 shows the bias of the wind speed intensity of the RCM versus reanalysis across the NEB for the austral winter (JJA) in the recent past period from 1986 to 2005. The captured wind speed patterns tend to be overestimated (between 1 and 3 m/s) in all simulations with the RCM RegCM4.7 (Figs 6A, 6B and 6C) in the NEB. The RegCM4.7_MPI (Fig 6B) overestimates throughout the state of MA, with velocities above 1 m/s, while the coastal strip between AL and BA presents bias between -1 and 1 m/s. The Remo2009_MPI (Fig 6I) is also a model that overestimates the wind speed throughout the NEB, with values that exceed 3 m/s on the north coasts of CE and RN. Simulations with the RCA4 model (Figs 6D, 6E, 6F, 6G, 6H) capture similar patterns for wind speed. The east coast from NEB from PB to BA is marked by bias between -1 and 1m/s, with small regions where velocities are underestimated by up to 2 m/s.

Fig 6. Bias of RCM wind speed intensity versus NEB-wide reanalysis for the austral winter (JJA).

Fig 7 shows the bias of the wind speed intensity of the RCM versus reanalysis across the NEB for the austral spring (SON) in the recent past period from 1986 to 2005. RCMs have increased wind speed for all regions of the NEB. In all models, the areas of overestimation were amplified spatially and with intensities between 1 and 3m/s. The eastern region of the NEB that goes from PB to the center of BA continues to be represented, similar to the JJA period by the simulations made with RCM RCA4 (Figs 7D, 7E, 7F, 7G, and 7H) and Remo2009 (Fig 7I). where the values of overestimation and underestimation do not exceed the absolute value of 1 m/s. The RegCM4.7_HadGEM2 model (Fig 7A) overestimates the winds throughout the NEB, with velocities above 1 m/s and some regions with values above 3 m/s.

Fig 7. Bias of RCM wind speed intensity versus NEB-wide reanalysis for austral autumn (SON).

Bias is a simple statistical metric made by subtracting the values between the model and the ECMWF-ERA5 reanalysis data. Thus, in order to make a more accurate study for results obtained, four study areas within the NEB region were selected (see areas in Fig 1). The monthly variability of wind speed in these areas will be analyzed through ECMWF-ERA5 reanalysis and soon after the models will be validated from the Taylor diagram.

Monthly variability of wind speed at 100 m from the ground in areas of the NEB.

Fig 8 shows the monthly distribution of wind speed from the ECMWF-ERA5 reanalysis at 100m from the ground in the NEB study areas, represented in Fig 1, in the recent past period from

1986 to 2005. All study areas have average wind speeds above 6 m/s (except C-BA) and may be favorable to the production of wind energy [6]. The N-CE and N-RN areas are coastal, so they have the highest velocity averages, with 6.78 and 7.39 m/s, respectively. The variance of wind speed is higher in the N-CE area, with a standard deviation of 1.77 m/s. One of the possible explanations for the greater variability in this area is associated with the seasonal migration of the ITCZ [36].

In addition, higher velocities recorded for these N-CE and N-RN areas, 9.38 and 9.75 m/s, respectively, during the austral spring, may be associated with marine and terrestrial breezes; and eastern trade winds and strengthening of southeast trade winds [6]. Marine and land breezes occur due to the temperature difference between land and water that warm and cool differently [37]. During the day, the continent warms faster than the water, generating a pressure gradient, in which the pressure on the continent is lower, blowing the wind from the ocean to the continent, called a sea breeze. At dusk, the continent cools faster than the ocean, and the pressure on it is greater, blowing the wind from the continent into the ocean, called the land breeze. Breezes can penetrate up to 100 km into the continent or ocean [38].

The easterly winds can also contribute to an increase in speed in the central region of the NEB, thus favoring the Borborema area to have a high average wind speed, with a value of 6.0 m/s. For this area, the maximum wind speed is 7.59 m/s in the austral spring and a minimum of 2.91 m/s in the austral autumn. The Borborema area is the one with the lowest standard deviation among all the study areas, with a value of 0.94 m/s.

In the C-BA area, winds of average intensity of 5.04 m/s may be associated with the high and flat topography, as well as the surface roughness that acts on the deceleration of the winds. [39] related the latitudinal position of the SASA and the wind intensity for Brazil, concluding a negative correlation throughout the year, indicating that when the SASA moves to lower latitudinal positions (during the austral winter) the intensity of the wind speed increases in the NEB, thus, maximum wind speeds of 7.26 m/s are recorded during this season of the year, while the austral summer registers a

minimum speed of 1.81 m/s. A possible explanation for the decrease in wind speed between spring and summer may be related to the passage of cold fronts in this area at the end of the year [40].

Fig 8. Monthly variability of wind speed at 100m from the ground in NEB areas: (a) northern Ceará (N-CE), (b) northern Rio Grande do Norte (N-RN), (c) Borborema Plateau between RN, PB and PE (Borborema) (d) central Bahia (C-BA), based on the ECMWF-ERA5 reanalysis for the recent past period from 1986 to 2005.

Validation: Regional Climate Models versus Reanalysis in NEB areas.

Fig 9 shows the monthly wind speed variability for each NEB study area. The models studied here are able to capture the pattern of the annual wind cycle produced by the reanalysis, although at times overestimating or underestimating. The models, in general, underestimate the wind speed for the first half of the year, and overestimate the wind speed for the second half of the year in the N-CE and N-RN areas. For the Borborema and C-BA areas, the periods of underestimation are shorter, restricted to the first four months of the year, while for the other months, the tendency is for overestimation in relation to the reanalysis. Despite the errors presented, the ability of the models to simulate the annual wind cycle in the areas indicates that they can be used for this research, and in studies of future projections.

Fig 9. Monthly wind speed variability (m/s) for reanalysis and RCMs for NEB areas (1986-2005). a) N-CE, b) N-RN, c) Borborema and d) C-BA

Fig 10 shows the Taylor diagrams for the study areas in the NEB. Here, all models are compared with each other for the specified areas. The best models for each area are those with the

highest correlation coefficient values, and the lowest standard deviation and RMSE values when compared with the ECMWF-ERA5 reanalysis.

For the N-CE area (Fig 10A), the best model is the RegCM4.7_HadGEM2, with a strong correlation higher than (0.80), standard deviation (2.50 m/s) and the second lowest RMSE (1.79 m/s) compared to the other models. Regarding the N-NB area (Fig 10B), most models present similar results, but the RCA4_EARTH model is far from the group of other models in the diagram. The best model is the RegCM4.7_HadGEM2, with a strong correlation (0.72), RMSE and standard deviation are similar to RegCM4.7_NorESM.

Regarding the Borborema area (Fig 10C), the RCA4_Miroc presented the lowest RMSE value (1.29 m/s), strong correlation (0.70) and low standard deviation (1.74 m/s), so it is indicated as the best representative model of wind speed in this area, RegCM4.7_NorESM also represents the area well, but presenting a moderate correlation (0.54).

In the C-BA area (Fig 10D), three models showed the best statistical indexes: RegCM4.7_NorESM, RCA4_miroc and RCA4_NorESM. The three models present Pearson's correlation classification as strong (0.72, 0.77 and 0.72). Regarding the standard deviation, the RCA4_Miroc and RCA4_NorESM models presented similar values (1.55 m/s), and the RMSE presented by the RCA4_Miroc was the lowest found (1.02 m/s). Thus, it was chosen in the C-BA area.

[41] evaluated the RCMs for Fortaleza (CE) for precipitation accumulation data in the period between 1970-2005. The authors found that the RegCM4.7_HadGEM2 model has good representativeness, being the third best evaluated model, the first two were not evaluated in this research. Thus, a better representation of this model for the wind speed compared to the rest could already be expected in this area of the N-CE. The RCA4_Miroc, obtained here as the best models in the Borborema and BA areas, was regularly evaluated for precipitation in the N-CE.

Fig 10. Taylor diagram between reanalysis and RCM for NEB areas in the recent past between 1986 and 2005. (a) N-CE area; (b) N-RN area; (c) Borborema area (d) C-BA area.

Wind power density from Regional Climate Models in NEB areas

Table 3 shows the amount of wind power density for the NEB areas in the recent past (1986 to 2005) from the RCM with the best statistical indices obtained. The area with the highest annual energy density is the N-RN with 4.963,4 W/m^2 per year, the N-CE has the second highest potential with 4.962,4W/m^2 per year, Borborema and central Bahia are, respectively, the third and fourth highest potential, with respectively, 2.240,5 W/m^2 per year and 2.184,4 W/m^2 per year. These last two regions have less than half the energy density of N-CE and N-RN.

As noted, the second half of the year presents a time of high wind power density in the NEB. 79,30% of the wind power density of the N-CE area is between the months of July and December, 72.01% for the N-RN area, 66,91% for the Borborema area and 59.67% in the C-BA area, this occurs mainly due to the action of the ITCZ and SASA weather systems that favor stronger winds in the second half of the year.

Table 3 Wind power density for NEB areas in the recent past between 1986 and 2005.

Months·of· the·year¤	(a)·N-CE¤	(b)·N-RN¤	(c)·Borborema¤	(d)·C-BA¤
January¤	226.3¤	240.0¤	38.8¤	48.6¤
February¤	90.0¤	100.7¤	29.5¤	55.3¤
March¤	81.6¤	125.2¤	53.9¤	78.2¤
April¤	72.3¤	131.7¤	125.1¤	180.3¤
May¤	130.4¤	257.7¤	217.9¤	245.5¤
June¤	426.7¤	533.9¤	276.1¤	272.9¤

July	600.2	569.6	287.3	273.3
August	736.3	665.2	320.9	302.9
September	848.5	724.4	296.7	277.8
October	766.5	671.4	257.8	239.1
November	607.8	555.3	222.5	146.2
December	375.8	388.3	114.0	64.3
Total	4.962,4W/m^{2}year	4.963,4W/m^{2}year	2.240,5W/m^2 year	2.184,4W/m^{2}year

Discussion

At first, the validation of the ECMWF-ERA5 reanalysis in relation to the observation was performed for the entire NEB, because it is a region that presents high natural climatic variability [42] caused by the action of atmospheric systems of different scales of time and space, such as: the Intertropical Convergence Zone (ITCZ), High-Level Cyclonic Vortices (HLV), Frontal Systems (SF), Eastern Wave Disturbances (EWD) and South Atlantic Convergence Zone (SACZ) [43,44], which may hinder the reproduction of specific wind patterns for this region.

The ECMWF-ERA5 reanalysis showed correlation coefficient values between moderate and strong, as well as bias on a scale between -1 and 1m/s, for most of the year, managing to capture the seasonal spatial pattern, making it suitable to be used as a reference in the validation of the climate of the recent past period of the RCMs belonging to the CORDEX-CORE. These results are in agreement with those obtained by [45], who also compared this reanalysis with data observed in the NEB and validated its use. It is important to note that the validation of the reanalysis in the cited study occur in a punctual manner, while the analysis of this research occurs in a spatial manner. This spatial validation was important because the ECMWF-ERA5 reanalysis presents dynamic data with wind speed outputs at 100 m from the ground, the ideal height for our study, since the current wind turbines are close to this height.

The seasonal evaluations between the RCMs and the ECMWF-ERA5 reanalysis showed that the RCMs are able to capture the wind speed patterns for the NEB region, however overestimating or underestimating values for most of this region, thus generating uncertainties for the evaluation of wind speeds in future scenarios. According to [18], bias errors can be characteristic of models, since the climate system is complex, dynamic, and non-linear where natural variabilities can affect the entire system, that is, the models carry uncertainties with them.

In addition, each RCM studied here receives large-scale forcings from different GCMs, so the parameters and physical input parameterizations from these GCMs contribute to produce different results between the RCMs. [46] classify that the quality of RCMs is associated with data from GCMs, described as "garbage in, garbage out". [47] stated that the empirical constants assigned to the physical parameterizations of the models are based on the observations of the domains in which they were developed, and can lead to uncertainties when transferred to other climatic regions. Thus, for more specific studies, it is necessary to search for the best data entry parameters for the NEB.

Another point to be highlighted, associated with the errors found in the models in relation to the reanalysis, is related to the representation of the topography of the NEB, which is considered a region with vast areas of complex topography. Thus, the spatial resolution of the models (mostly within 50 km) may not be able to capture the topographic representation in complex locations. In addition, it is worth mentioning that the wind speed produced by the RCA4 and Remo2009 models were extrapolated via logarithmic equations, which according to [8], from this technique, the data carry uncertainties due to its dependence on the surface roughness.

As for atmospheric phenomena, the ITCZ is characterized by a vast area of atmospheric instability and formation of large convective clouds along the equatorial region [48]. The atmospheric system acts in the northern portion of the NEB (when it is located further south) mainly in the austral autumn (MAM). This intensification of the ITCZ (further south) is more evident in the models: RegCM4.7_HadGEM2 (Fig 5A), RCA4_HadGEM2 (Fig 5D), Remo2009_MPI (Fig 5I) that overestimated the wind speed in the northern region of the NEB in DJF and showed a decrease in the

intensity of the bias and the overestimated area in MAM compared to the reanalysis. Models RegCM4.7_NorESM, RCA4_NorESM and RCA4_Miroc (Figs 5C, 5F and 5H) show a shifted ITCZ further north when compared to the MAM reanalysis, since the areas that underestimated decreased in area and intensity in relation to DJF.

In the obtained results, the GCM: MPI and NorESM showed similar patterns when applied in different RCMs. For the RegCM4.7_MPI, RCA4_MPI and REMO2009_MPI models, similar wind speed patterns were observed for all seasons, the same occurred for the RegCM4.7_NorESM and RCA4_NorESM models. The GCM here called EARTH when associated with the RCM RCA4 captured wind speed patterns similar to those of the GCM MPI (RegCM4.7_MPI, RCA4_MPI and Remo2009_MPI). The GCM Miroc when associated with the RCM RCA4 captured wind patterns similar to the GCM NorESM (especially the RCA4_NorESM), this greater similarity may be associated with the fact that the parameters used in the RCM are the same. The models that received the GCM HadGEM2 picked up different patterns resembling each other only in the austral spring.

In this research, nine RCMs were studied separately. The bias results ranged from -3 to 3 m/s, with some regions exceeding this range of values. These results are similar to those of [10], it is noteworthy that the study developed by [10], used an ensemble of models by arithmetic means to reduce uncertainties and compare with the reanalysis of the Era-Interim for the recent past period (1979-2005). Therefore, in this research, even though there was no ensemble of models, the results found are satisfactory, as they present the same margin of error. In general, the models tended to overestimate the NEB region for almost its entirety at the JJA and SON stations, which are the most promising months for wind power production. Thus, when making future projections with these models, values above expectations can be generated when compared to the reanalysis.

Regarding the seasonality of wind speed for the chosen four areas in NEB, it is well defined in the study areas, with the median of the velocities increasing in April, peaking in September and then declining. In addition, this seasonality plays a very important role in the production of electricity for the NEB region. As seen in Figs (8A, 8B, 8C and 8D), wind speeds become more intense for the

JJA and SON seasonal periods. While during this period, part of the NEB states tend to have a period of drought [5], one of the exceptions is the area of the east coast of the NEB, which includes a part of the N-RN area (Fig 8B) that, due to the action of the EWD [49], cause rainfall during the austral winter. This relationship between wind speed and precipitation is important for the study of hydro-wind complementarity in the NEB region. In addition, for the austral winter (JJA), the SASA moves west from the South Atlantic during this period, hindering convective movements and cold fronts in southeastern Brazil, decreasing precipitation [50]. Thus, hydroelectric plants operate with low water levels, requiring a complementary source of energy.

Wind energy presents itself as a clean and complementary source to the hydroelectric plant, this complementarity is important for the country. Therefore, identifying good wind energy producing areas becomes indispensable. For this, it is important to minimize possible errors by choosing the RCM that best represents the wind speed for the study area, during the period of analysis in the recent past from 1986 to 2005.

The RCMs were analyzed for the areas and despite the investments made by institutions to improve RCMs, critical biases are still found in several studies [10,51,52]. In addition, a high tendency to overestimate the models regarding reanalysis has also been found in several studies [52-54]. The results presented by the Taylor Diagram were satisfactory, since for all areas of study, all the models studied present correlation values above 0.7, which indicates that the phases of the annual wind speed cycles for each region are well represented by the models. On the other hand, the RMSE shows a tendency of similarity between the simulation of the annual wind speed cycles by the models when compared with the reanalysis. The values for the RMSE of the best models vary between 1.02 m/s (C-BA) and 2.16 m/s (N-CE); these values probably arise from the high overestimation values for the JJA and SON stations. In addition, the analysis of the Taylor diagram also shows that in different areas, different models present superior results, and not a single model performs better than the other in different areas of the NEB.

Finally, the calculated values of wind power density has show that the NEB region is conducive to wind energy production. Knowing the wind power density is important to identify areas where the wind is strong and constant enough to generate electricity effectively and economically, as well as helping in planning the energy demand, which can be met by this energy source. The results show that between 59% and 80% of the wind power density corresponds to the second half of the year in Brazil, this time of which consists of low amounts of rainfall. In this way, wind energy serves as a complementary energy source to hydroelectric power for this time. It is important to highlight that for better use of wind energy in the NEB, more in-depth studies need to be carried out, considering, for example, the roughness of the areas to be explored as well as the types of turbines appropriate for each location.

Conclusion

The objective of this research was to calculate the density of wind power from regional climate models in areas in the Northeast of Brazil (NEB) during the period from 1986 to 2005, the recent past. It is one of the first studies with a full focus on NEB, comparing nine RCM with the newest ECMWF-ERA5 reanalysis. In addition, it studies areas that have large numbers of wind farms, where the best models for each study area were identified, wind speed was analyzed and wind power density was calculated in the recent past.

In this research, the reanalysis of the ECMWF-ERA5 was validated in relation to the data observed through the meteorological variable wind speed at 10m from the ground. The results showed bias between -1 and 1 m/s for most of the NEB in all seasonalities and larger regions with moderate and strong correlation.

In the validation of the RCM participating in the CORDEX-CORE project, the models studied tended to overestimate or underestimate the wind speed compared to the reanalysis at 100m height for all seasons of the year in all regions of the NEB. There is a strong overestimation of the winds in

the second half of the year, and for the first half of the year, the errors are minimized coinciding with the rainy season.

The behavior of wind speed at 100 m height from the ground was also observed. The coastal regions presented the highest records of average and maximum speed and also presented greater variability in wind speed, being associated with their geographical position, as well as by the low roughness of the soil. All the areas delimited for study in the NEB are considered good producers of wind energy and, therefore, are the areas that currently have the largest number of installed wind farms.

When evaluated for the NEB areas, the RCMs that presented statistical metrics closest to the reanalysis were: RegCM4.7_HadGEM2 (N-CE and N-RN), RCA4-Miroc (Borborema and C-BA). This demonstrates that the dynamic downscaling technique promotes RCMs that can represent one area better than another, especially in areas that present a large number of climate phenomena.

Finally, the wind power density was calculated for the recent past from 1986 to 2005. It was observed that the N-RN region was the one with the highest wind potential, while the central region of Bahia had the lowest potential among the areas studied. The coastal regions of Ceará and Rio Grande do Norte had more than double the wind potential when compared to the other areas studied.

This research offered an analysis of the behavior of wind speed in this region, thus providing a scientific basis for wind power generation development policies contributing to the reduction of GHG emissions. It is believed that with good strategic planning, this type of energy can grow in the future and reduce the country's dependence on hydroelectric sources, also alleviating the problems of generating electricity in times of drought.

Reference

[1] Gao Y, Ma S, Wang T. The impact of climate change on wind power abundance and variability in China. Energy. 2019;189:116215. https://doi.org/10.1016/j.energy.2019.116215.

[2] Owusu PA, Asumadu-Sarkodie S. A review of renewable energy sources, sustainability issues and climate change mitigation. Cogent Engineering. 2016;3:1167990. https://doi.org/10.1080/23311916.2016.1167990.

[3] Hamed TA, Alshare A. Environmental impact of solar and wind energy- A review. Journal of Sustainable Development of Energy, Water and Environment Systems. 2021;10:1–23. https://doi.org/10.13044/j.sdewes.d9.0387.

[4] IRENA. Monthly Electricity Statistics. 2023. Available from: https://www.iea.org/data-and-statistics/data-tools/monthly-electricity-statistics. Accessed in: 12 jan 2023

[5] Marengo JA, Torres RR, Alves LM. Drought in Northeast Brazil—past, present, and future. Theor Appl Climatol. 2017;129:1189–1200. https://doi.org/10.1007/s00704-016-1840-8.

[6] Amarante OAC, Brower M, Zack J, Sá AL. Atlas do Potencial Eólico Brasileiro. Brasília: Ministério de Minas e Energia—ELETROBRÁS; 2001. Available from: http://www.cresesb.cepel.br/index.php?section=publicacoes&task=livro&cid=1. Accessed in: 12 fev 2023.

[7] Associação Brasileira de Energia Eólica. Boletim Mensal de Geração Eólica. 2023. Available from: https://abeeolica.org.br/wp-content/uploads/2023/06/Boletim-de-Geracao-Eolica-202 2.pdf. Accessed in: 02 mar 2023

[8] Emeksiz C. Multi-gen genetic programming based improved innovative model for extrapolation of wind data at high altitudes, case study: Turkey. Comput Electr Eng. 2022;100:107966. https://doi.org/10.1016/j.compeleceng.2022.107966.

[9] Hadi FA. Diagnosis of the Best Method for Wind Speed Extrapolation. International Journal of Advanced Research in Electrical, Electronics and Instrumentation Engineering. 2015;4:8176-8183. https://doi.org/10.15662/IJAREEIE.2015.0410058.

[10] Reboita MS, Amaro TR, de Souza MR. Winds: intensity and power density simulated by RegCM4 over South America in present and future climate. Clim Dyn. 2018;51:187–205. https://doi.org/10.1007/s00382-017-3913-5.

[11] Gil Ruiz SA, Barriga JEC, Martínez JA. Wind power assessment in the Caribbean region of Colombia, using ten-minute wind observations and ERA5 data. Renewable Energy. 2021;172:158–176. https://doi.org/10.1016/j.renene.2021.03.033.

[12] Slivinski LC. Historical reanalysis: What, how, and why? J Adv Model Earth Syst. 2018;10:1736–1739. https://doi.org/10.1029/2018ms001434.

[13] Hersbach H, Bell B, Berrisford P, et al. The ERA5 global reanalysis. Quart J Roy Meteor Soc. 2020;146:1999–2049. https://doi.org/10.1002/qj.3803.

[14] Staffell I, Pfenninger S. Using bias-corrected reanalysis to simulate current and future wind power output. Energy. 2016;114:1224–1239. https://doi.org/10.1016/j.energy.2016.08.068.

[15] Molina MO, Gutiérrez C, Sánchez E. Comparison of ERA5 surface wind speed climatologies over Europe with observations from the HadISD dataset. Int J Climatol. 2021;41:4864–4878. https://doi.org/10.1002/joc.7103.

[16] Giorgi F, Jones C, Asrar GR, et al. Addressing climate information needs at the regional level: the CORDEX framework. WMO Bull. 2009;58:175.

[17] World Climate Research Programme. Evaluating and Improving Regional Climate Projections. http://wcrp.ipsl.jussieu.fr/Workshops/Downscaling/Documents/CORDEX_Workshop0 9.pdf. Accessed 03 April 2023.

[18] Ambrizzi T, Reboita MS, da Rocha RP, Llopart M. The state of the art and fundamental aspects of regional climate modeling in South America. Ann N Y Acad Sci. 2019;1436:98–120. https://doi.org/10.1111/nyas.13932.

[19] Xavier AC, King CW, Scanlon BR. Daily gridded meteorological variables in Brazil (1980-2013). Int J Climatol. 2016;36:2644–2659. https://doi.org/10.1002/joc.4518.

[20] Elguindi N, Bi X, Giorgi F, Nagarajan B, Pal J, Solomon F, et al. Regional Climate Model RegCM Reference Manual Version 4.7. Trieste, Italy: The Abdus Salam International Centre for Theoretical Physics; 2017.

[21] Samuelsson P, Jones CG, Willén U, et al. The Rossby Centre Regional Climate model RCA3: model description and performance. Tellus A. 2011;63:4. https://doi.org/10.1111/j.1600-0870.2010.00478.x.

[22] Jacob D, Elizalde A, Haensler A, et al. Assessing the Transferability of the Regional Climate Model REMO to Different COordinated Regional Climate Downscaling EXperiment (CORDEX) Regions. Atmosphere. 2012;3:181–199. https://doi.org/10.3390/atmos3010181.

[23] Jones CD, Hughes JK, Bellouin N, et al. The HadGEM2-ES implementation of CMIP5 centennial simulations. Geosci Model Dev. 2011;4:543–570. https://doi.org/10.5194/gmd-4-543-2011.

[24] Bentsen M, et al. The Norwegian Earth System Model, NorESM1-M – Part 1: Description and basic evaluation. Geosci Model Dev. 2012;5:2843-293. https://doi.org/10.5194/gmdd-5-2843-2012.

[25] Giorgetta MA, Jungclaus J, Reick CH, et al. Climate and carbon cycle changes from 1850 to 2100 in MPI-ESM simulations for the Coupled Model Intercomparison Project phase 5. J Adv Model Earth Syst. 2013;5:572–597. https://doi.org/10.1002/jame.20038.

[26] Koenigk T, Brodeau L, Graversen RG, et al. Arctic climate change in 21st-century CMIP5 simulations with EC-Earth. Clim Dyn. 2013;40:2719–2743. https://doi.org/10.1007/s00382-012-1505-y.

[27] Watanabe M, Suzuki T, O'ishi R, et al. Improved Climate Simulation by MIROC5: Mean States, Variability, and Climate Sensitivity. J Clim. 2010;23:6312–6335. https://doi.org/10.1175/2010JCLI3679.1.

[28] Custódio RS. Energia eólica para produção de energia elétrica. Rio de Janeiro: Eletrobrás; 2013.

[29] Elguindi N, Bi X, Giorgi F, et al. Regional climate model RegCM user manual version 4.4. Trieste, Italy: The Abdus Salam International Centre for Theoretical Physics; 2013

[30] Wilks DS. Statistical Methods in the Atmospheric Sciences. Academic Press; 2011.

[31] Devore JL. Probabilidade e Estatística para Engenharia e Ciências. Cengage Learning Edições Ltda; 2006.

[32] Taylor KE. Summarizing multiple aspects of model performance in a single diagram. J Geophys Res D: Atmos. 2001;106:7183-7192. https://doi.org/10.1029/2000JD900719.

[33] Sawadogo W, Reboita MS, Faye A, et al. Current and future potential of solar and wind energy over Africa using the RegCM4 CORDEX-CORE ensemble. Clim Dyn. 2021;57:1647–1672. https://doi.org/10.1007/s00382-020-05377-1.

[34] Diaconita A, Onea F, Rusu E. An evaluation of the wind energy in the North Sea coast. Mechanical Testing and Diagnosis. 2019;9:17–22. https://doi.org/10.35219/mtd.2019.1.02.

[35] Wallace JM, Hobbs PV. Atmospheric Science: An Introductory Survey. Elsevier. 2006.

[36] Cavalcanti IFA. Tempo e clima no Brasil. Oficina de Textos; 2009.

[37] Natalia F. Meteorologia Sinótica. Universitária/UFPel; 2001.

[38] Ferreira AG, Mello NGS. Principais sistemas atmosféricos atuantes sobre a região Nordeste do Brasil e a influência dos oceanos Pacífico e Atlântico no clima da região. Rev Bras Climatol. 2005. https://doi.org/10.5380/abclima.v1i1.25215.

[39] Gilliland JM, Keim BD. Surface wind speed: trend and climatology of Brazil from 1980-2014. Int J Climatol. 2018;38:1060–1073. https://doi.org/10.1002/joc.5237.

[40] Kousky VE. Frontal Influences on Northeast Brazil. Mon Weather Rev. 1979;107:1140–1153. https://doi.org/10.1175/1520-0493(1979)1072.0.CO;2.

[41] Silva AEF, et al. Desempenho das Simulações do Projeto Cordex Quanto à Representação dos Padrões de Variação da Precipitação no Século XX sobre o Município de Fortaleza, Ceará. Rev Bras Meteorol. 2020;35:387-396. https://doi.org/10.1590/0102-7786353003.

[42] Hastenrath S. Interannual Variability and Annual Cycle: Mechanisms of Circulation and Climate in the Tropical Atlantic Sector. Mon Weather Rev. 1984;112:1097–1107. https://doi.org/10.1175/1520-0493(1984)1122.0.CO;2.

[43] Uvo CB, Repelli CA, Zebiak SE, Kushnir Y. The Relationships between Tropical Pacific and Atlantic SST and Northeast Brazil Monthly Precipitation. J Clim. 1998;11:551–562. https://doi.org/10.1175/1520-0442(1998)0112.0.CO;2.

[44] Molion LCB, Bernardo SO. Uma revisão da dinâmica das chuvas no nordeste brasileiro. Rev Bras Meteorol. 2002;17:1-10.

[45] Siefert CAC, Dombrowski Netto N, Marangon FHS, et al. Avaliação de Séries de Velocidade do Vento de Produtos de Reanálises Climáticas para o Brasil. Rev bras meteorol. 2022;36:689–701. https://doi.org/10.1590/0102-7786360026.

[46] Giorgi F, Mearns LO. Approaches to the simulation of regional climate change: A review. Reviews of Geophysics. 1991;29:191. https://doi.org/10.1029/90rg02636.

[47] Takle ES, Roads J, Rockel B, et al. Transferability Intercomparison: An Opportunity for New Insight on the Global Water Cycle and Energy Budget. Bull Am Meteorol Soc. 2007;88:375–384. https://doi.org/10.1175/BAMS-88-3-375.

[48] Utida G, Cruz FW, Etourneau J, et al. Tropical South Atlantic influence on Northeastern Brazil precipitation and ITCZ displacement during the past 2300 years. Sci Rep. 2019;9:1698. https://doi.org/10.1038/s41598-018-38003-6.

[49] Yamazaki Y. Estudos teóricos e sinóticos dos distúrbios tropicais. INPE; 1975.

[50] Reboita MS, Ambrizzi T, Silva BA, et al. The south Atlantic subtropical anticyclone: Present and future climate. Front Earth Sci. 2019. https://doi.org/10.3389/feart.2019.00008.

[51] Yang J, Astitha M, Monache LD, Alessandrini S. An Analog Technique to Improve Storm Wind Speed Prediction Using a Dual NWP Model Approach. Mon Weather Rev. 2018;146:4057–4077. https://doi.org/10.1175/MWR-D-17-0198.1.

[52] Libanda B. Performance assessment of CORDEX regional climate models in wind speed simulations over Zambia. Modeling Earth Systems and Environment. 2023;9:253–262. https://doi.org/10.1007/s40808-022-01504-5.

[53] Kulkarni S, Deo MC, Ghosh S. Performance of the CORDEX regional climate models in simulating offshore wind and wind potential. Theor Appl Climatol. 2019;135:1449–1464. https://doi.org/10.1007/s00704-018-2401-0.

[54] Wu J, Han Z-Y, Yan Y-P, et al. Future changes in wind energy potential over China using RegCM4 under RCP emission scenarios. Advances in Climate Change Research. 2021;12:596–610. https://doi.org/10.1016/j.accre.2021.06.005.

Fig1

Click here to access/download;Figure;Fig 1.tif

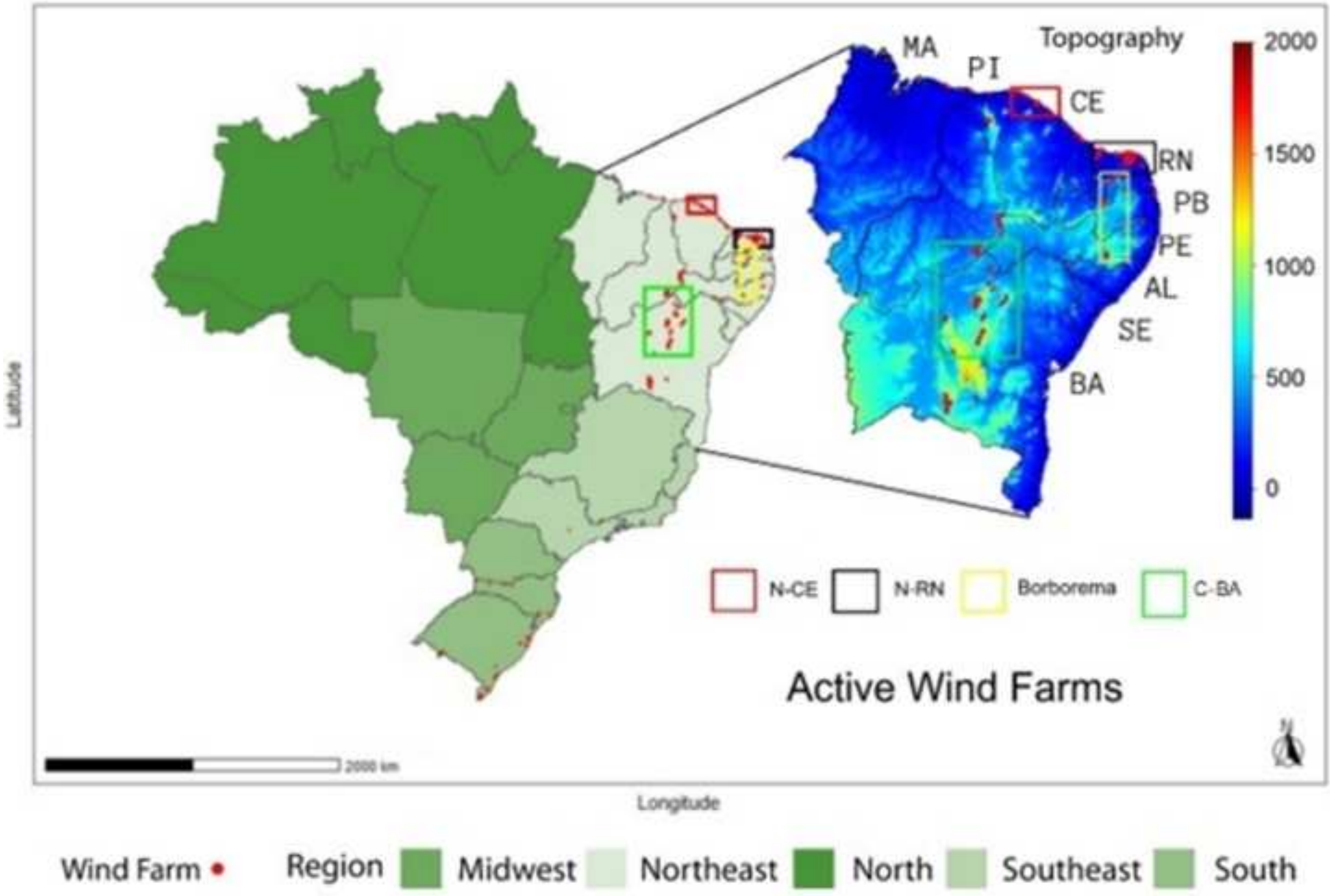

Fig2

Click here to access/download;Figure;Fig 2.tif

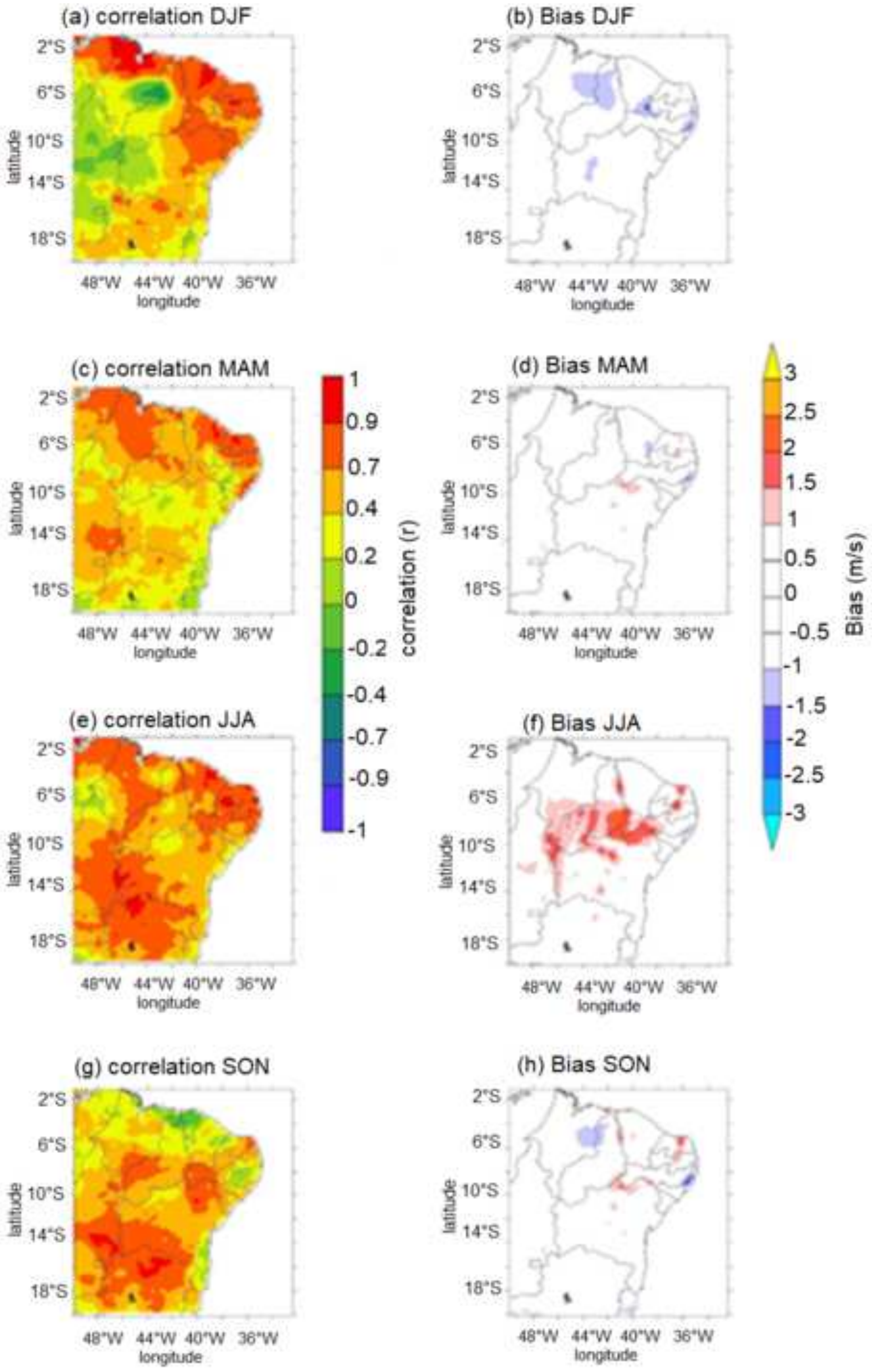

Fig3

Click here to access/download;Figure;Fig 3.tif

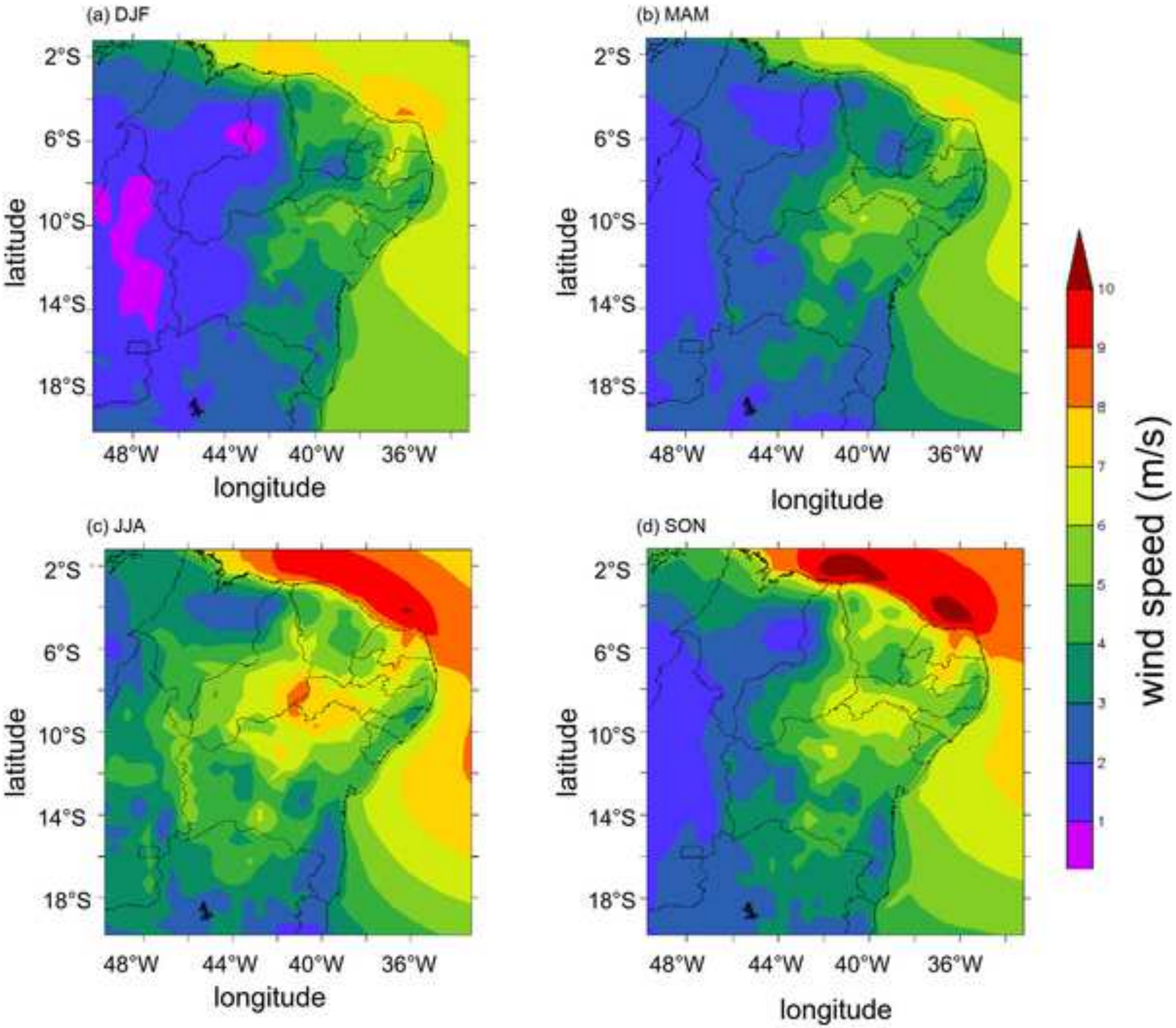

Fig4

Click here to access/download;Figure;Fig 4.tif

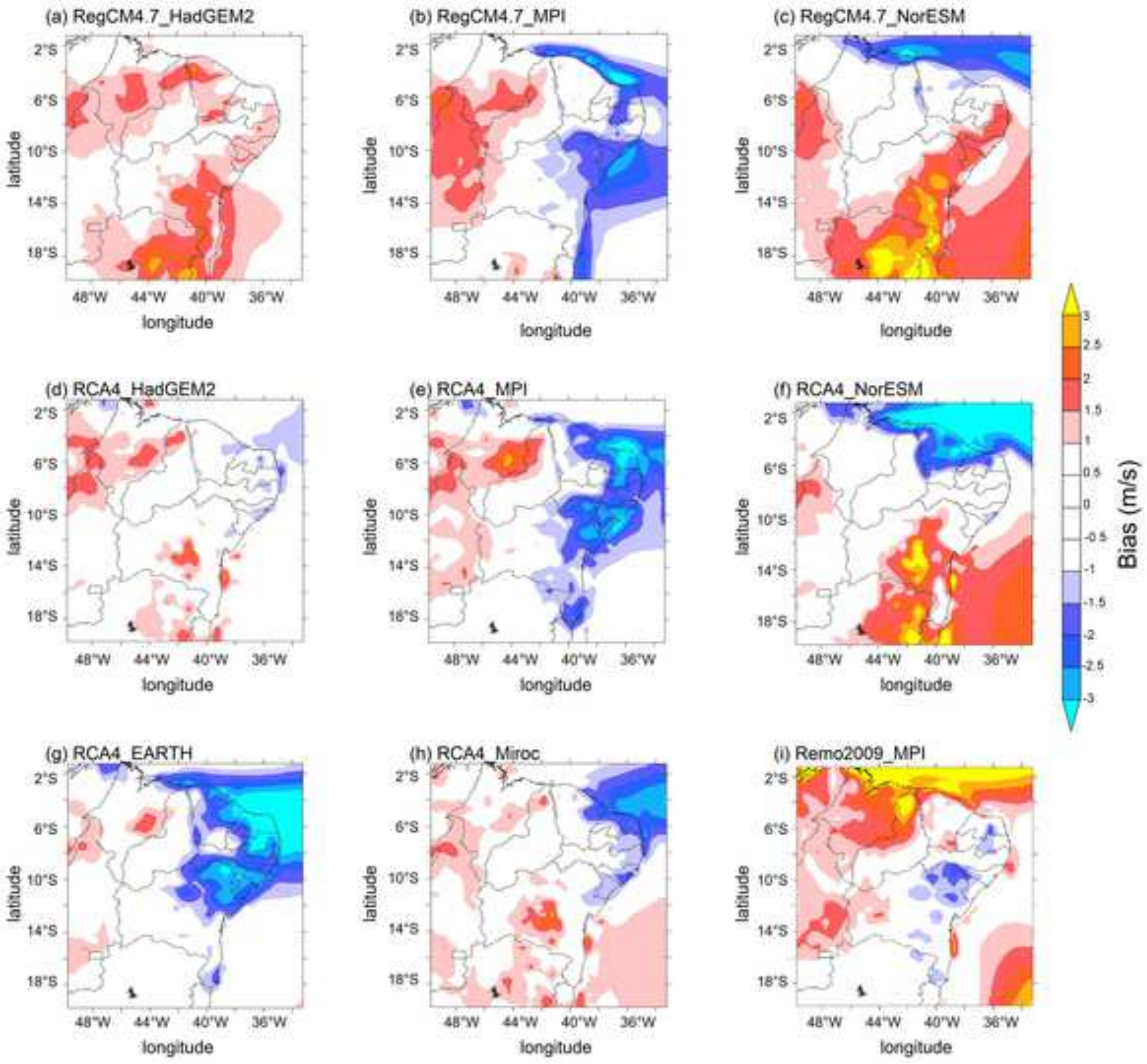

Fig5

Click here to access/download;Figure;Fig 5.tif

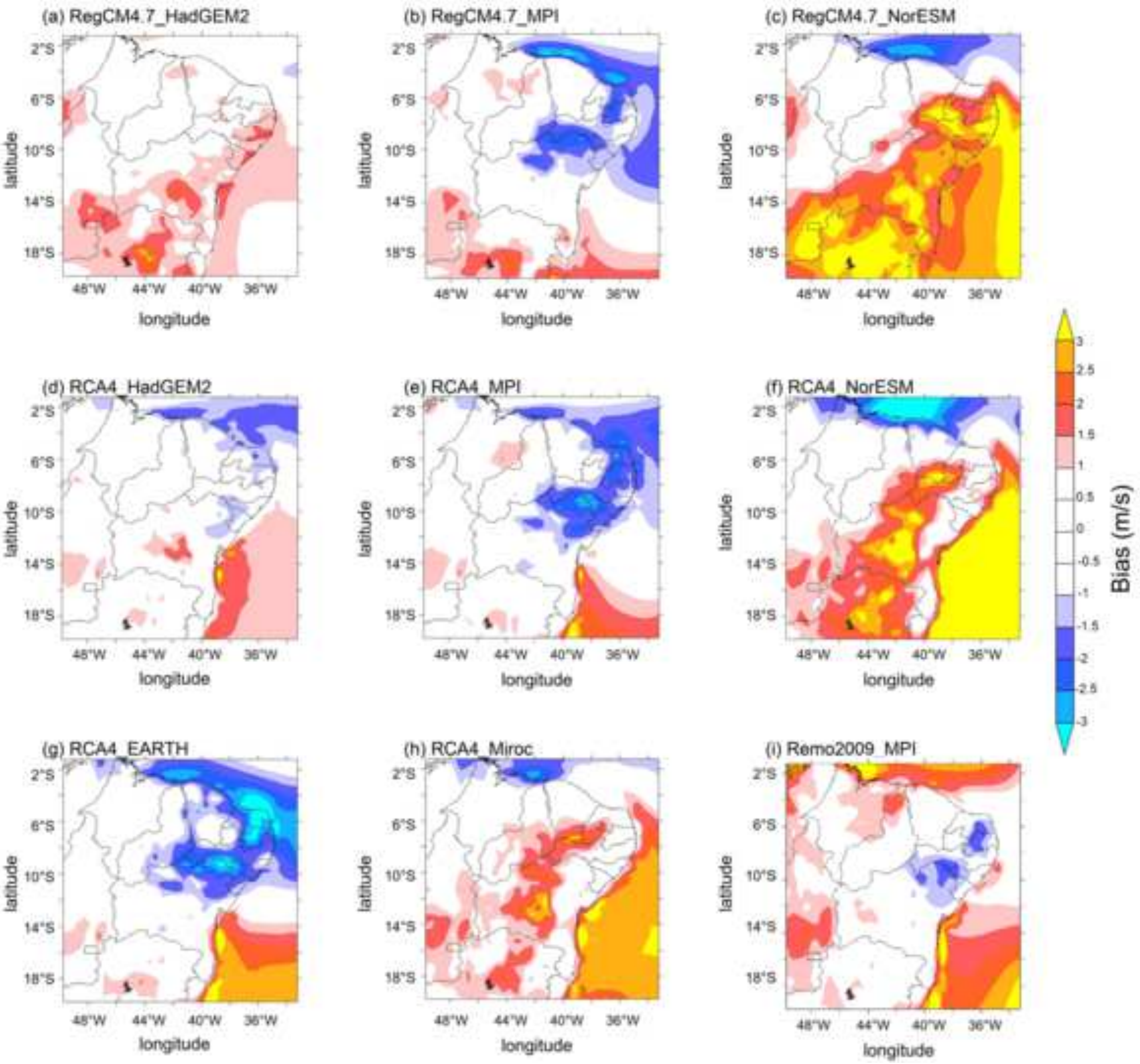

Fig6

Click here to access/download;Figure;Fig 6.tif

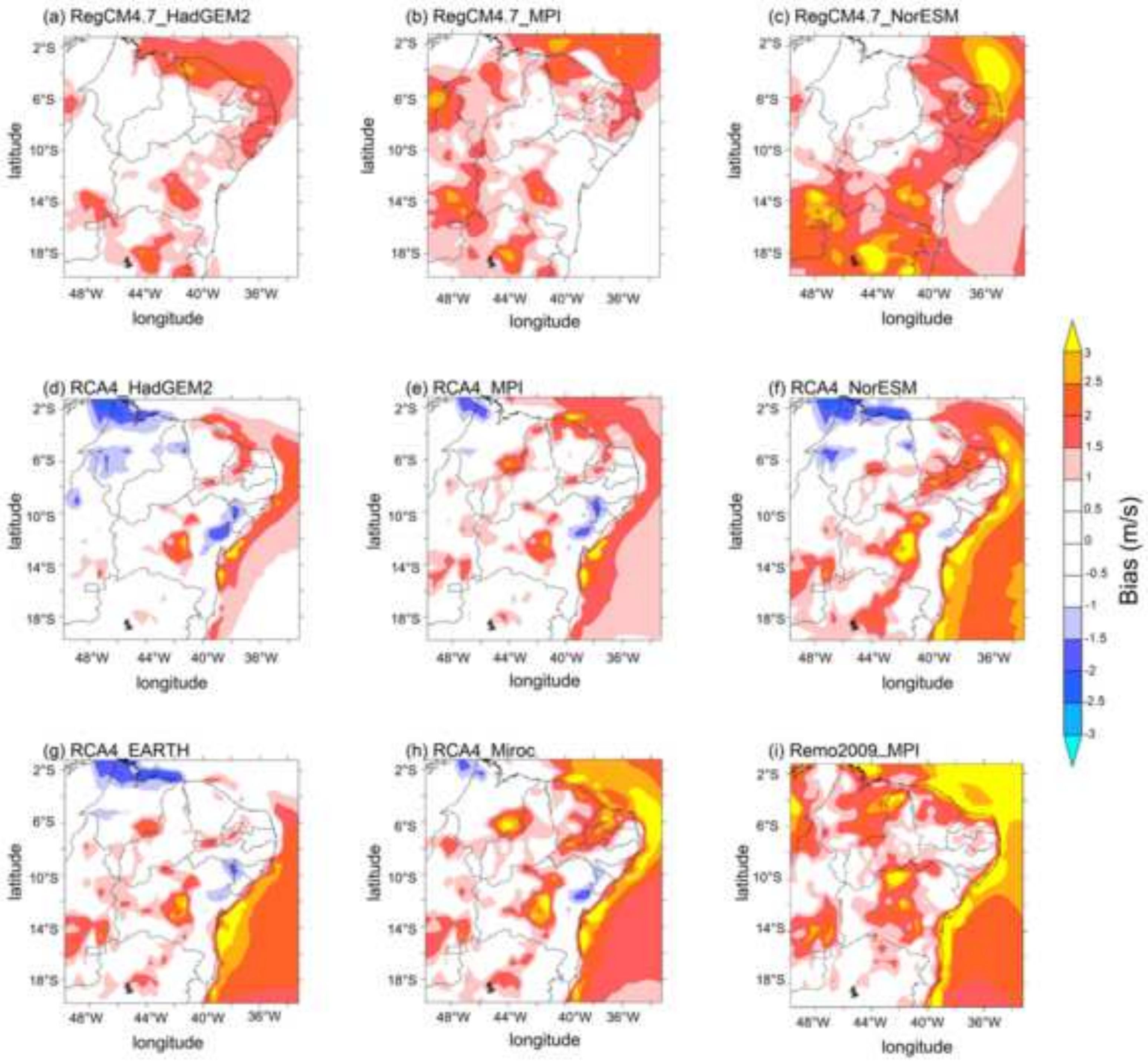

Fig7

Click here to access/download;Figure;Fig 7.tif

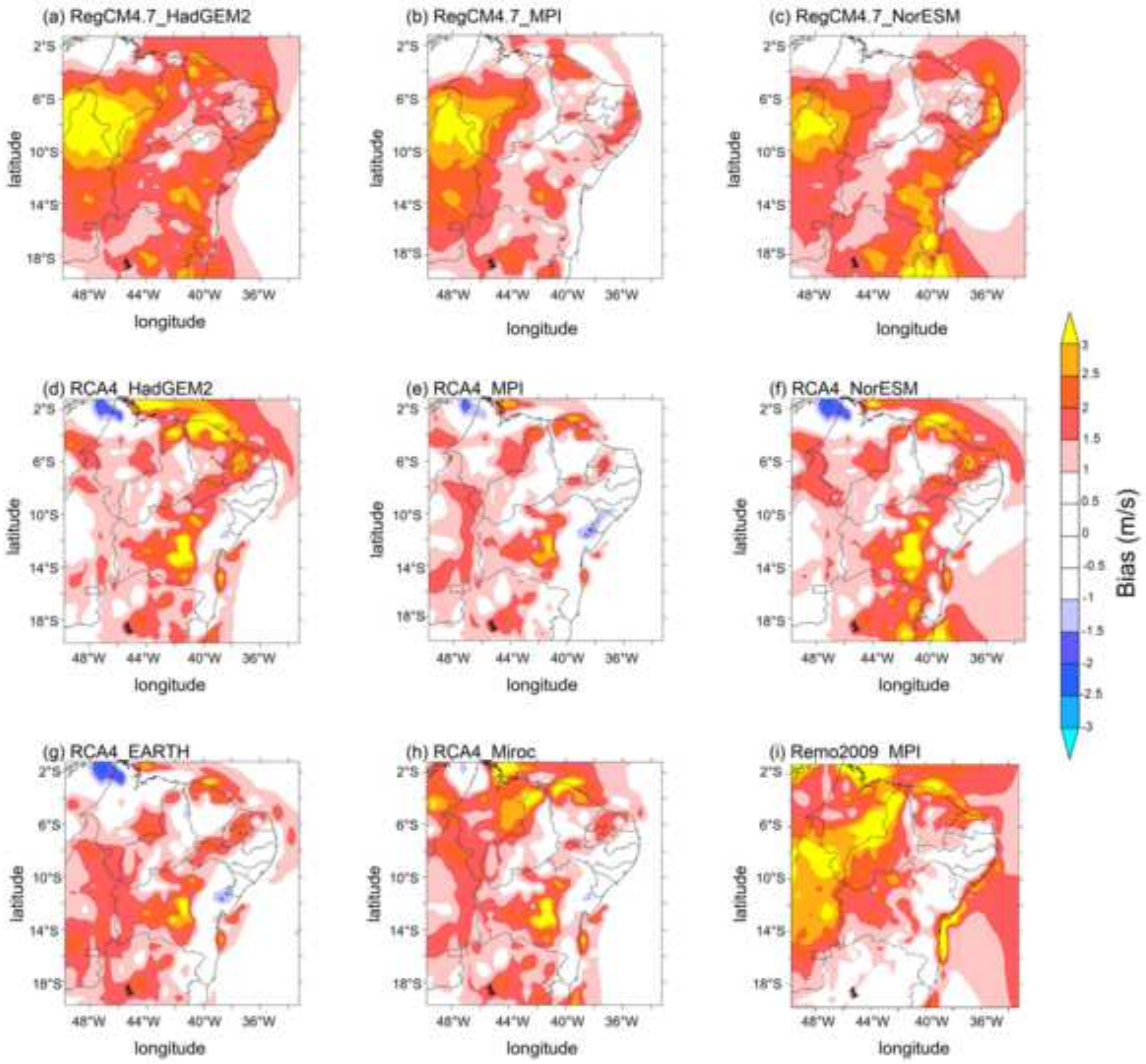

Fig8

Click here to access/download;Figure;Fig 8.tif

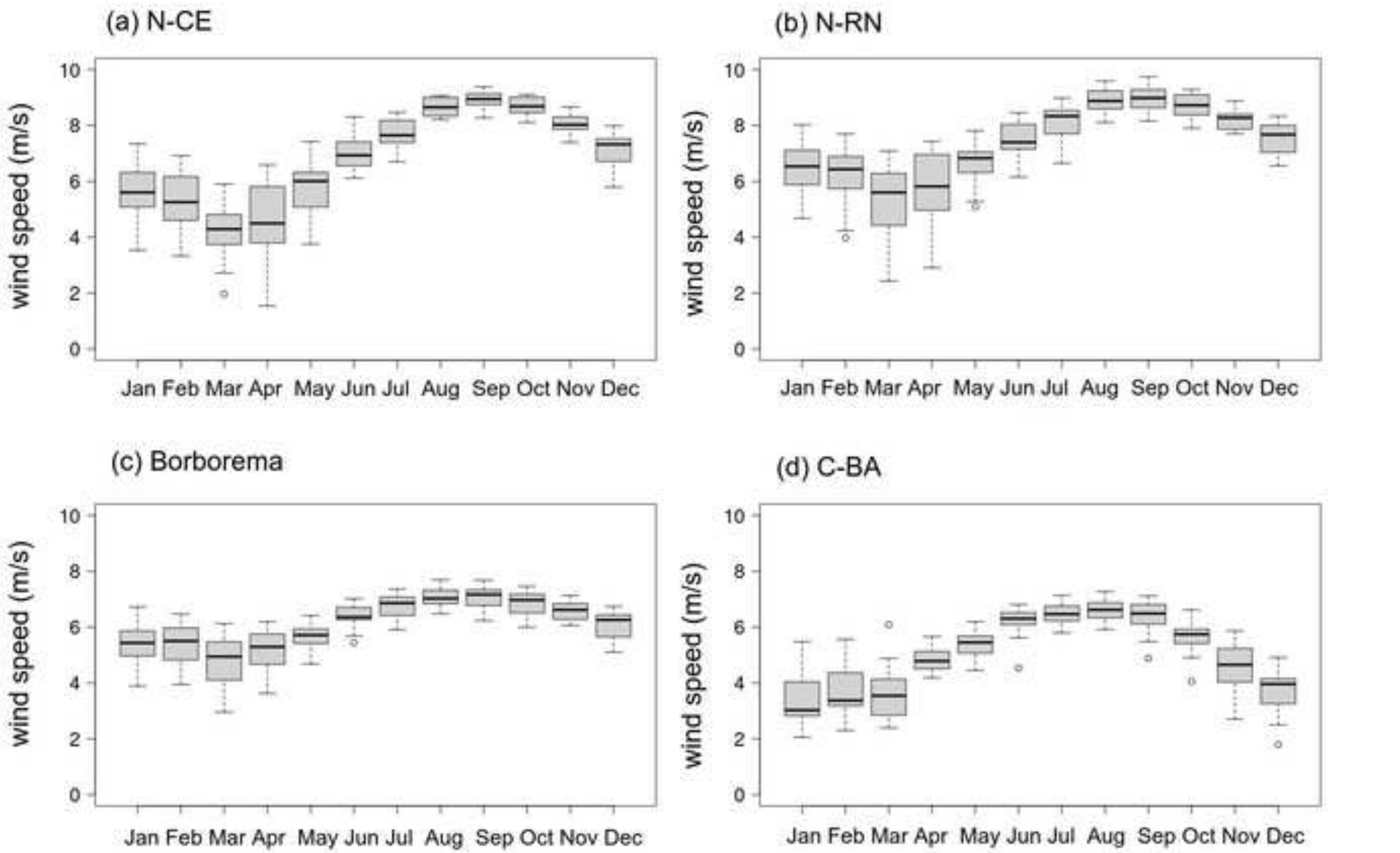

Fig9

Click here to access/download;Figure;Fig 9.tif

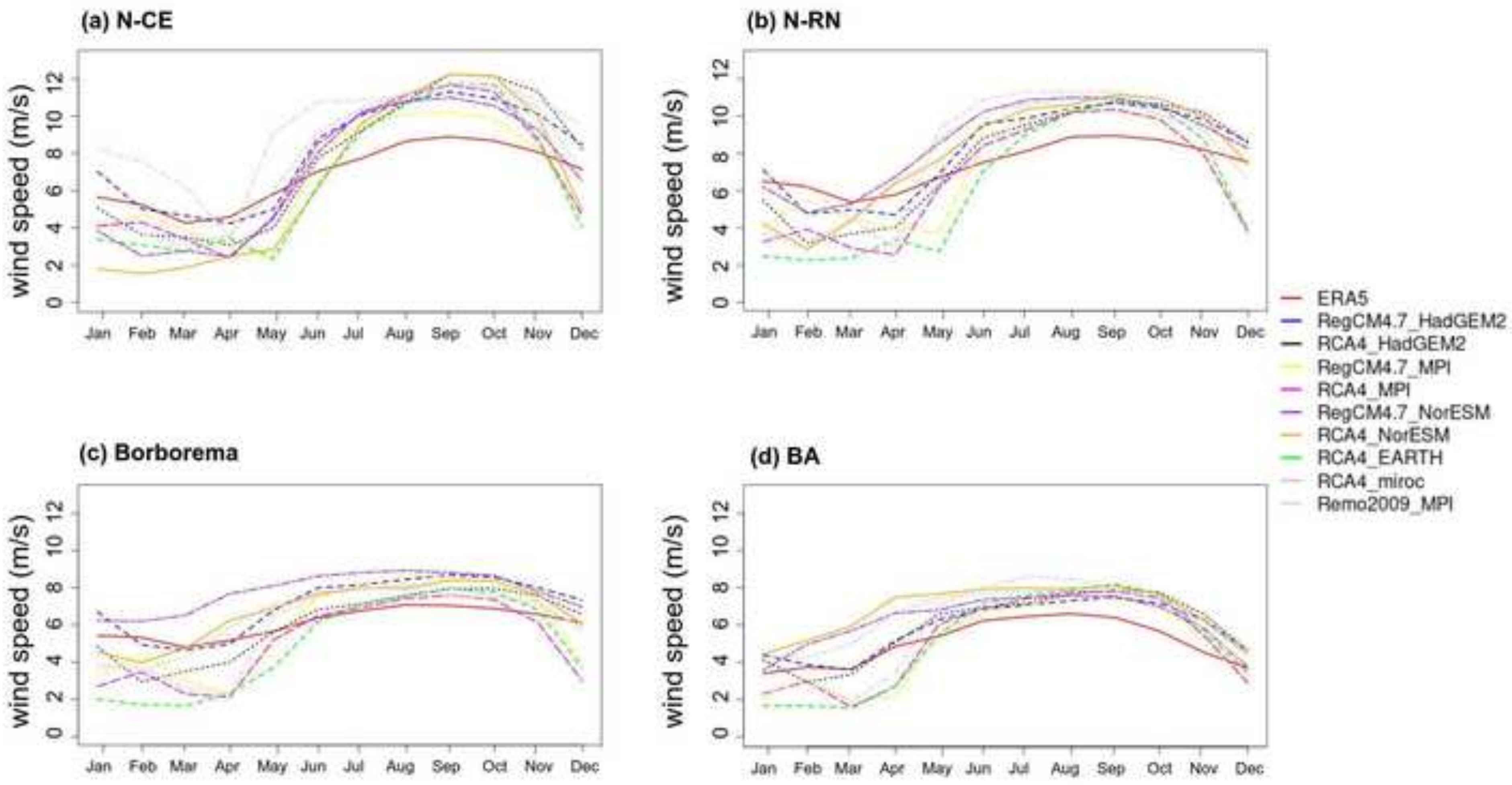

Fig10

Click here to access/download;Figure;Fig 10.tif

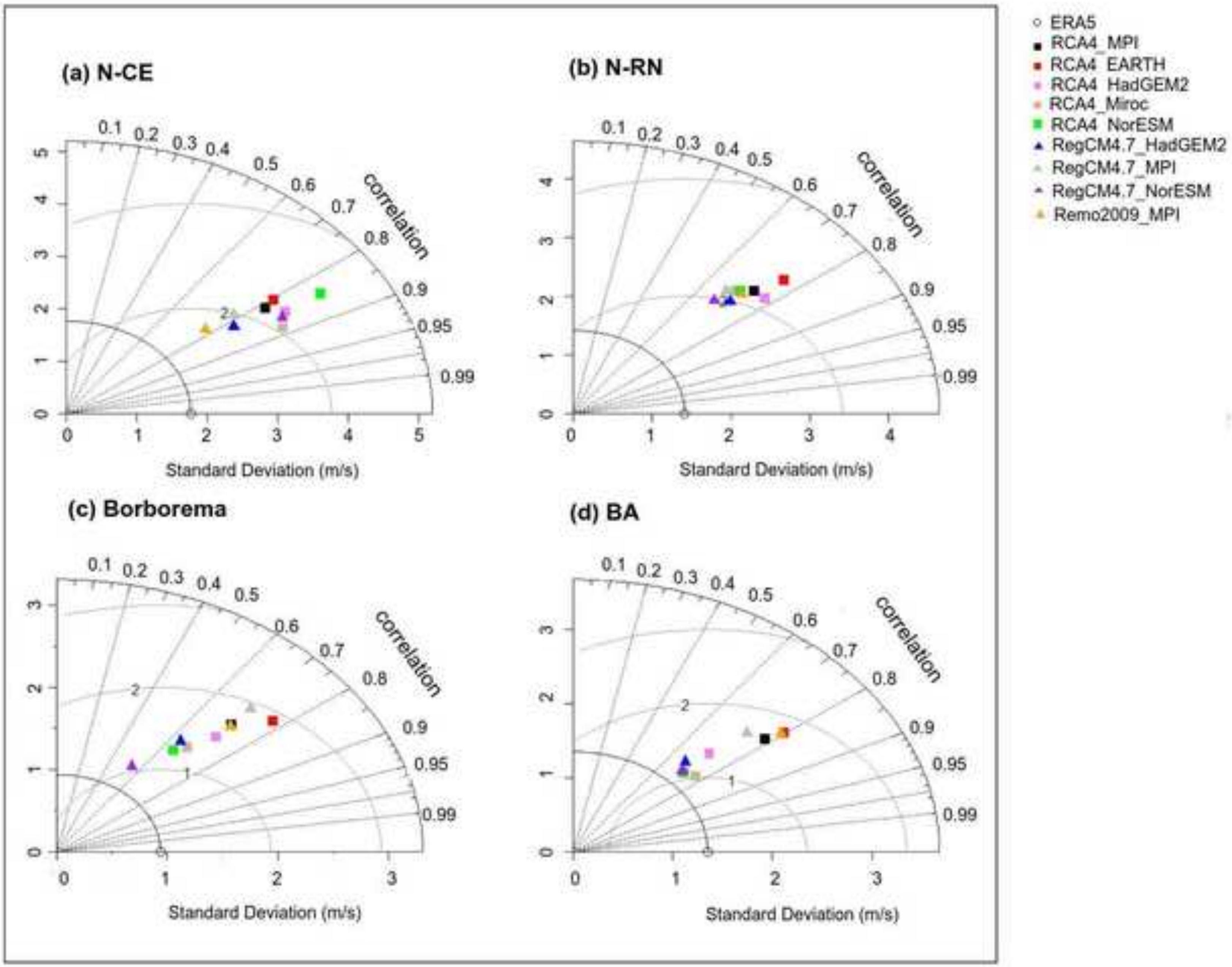

Supplement: Attachment — Submitted filename: PONE-D-24-02304_reviewer.pdf [file pone.0307641.s001.pdf]
