# Supplementary material for: Wind power density in areas of Northeastern Brazil from Regional Climate Models for a recent past
Source: PLoS One. 2024 Jul 25;19(7):e0307641. doi: 10.1371/journal.pone.0307641 (PMC11271907; doi:10.1371/journal.pone.0307641)
Supplement: Attachment — Submitted filename: Response_to_reviews_Augusto_plos_one.docx [file pone.0307641.s003.docx]

**Wind power density in areas of Northeastern Brazil from regional climate models for a recent past**

Answers

Reviewer #2

1) Although my suggestions were incorporated into the manuscript and the English was improved, the text still needs some language verification.

Answer: Thanks! The English was improved. If needs more improve, please select the sentences.

2) some discussion/explanation about the following topic:

   -> is the variability not important for a wind farm? This question was firstly posed in the context of the use of monthly data information against high-frequency variability. A paragraph on that could be included in the discussion section. Please note that this topic can also be directly related to a second aspect mentioned in the cover letter and included into the text: "Reanalysis provides the average of the variable over a time interval, impacting the estimation of maximum and minimum values.", which is related to the relative low performance of ERA5 in SON. So, there is some missing variability in the high frequency as well as in the monthly averages. How do manage with that when considering future projection, for instance?

Answer: A paragraph was written in conclusion section.

3) Moreover, equation 8 is not correct, simply because the denominator should be (273.15 + T), I mean, the "T" is not added to the prior fraction, being is part of its denominator. Please correct.

Answer: The equation was corrected
